# Conserved patterns of transcriptional dysregulation, heterogeneity, and cell states in clear cell kidney cancer

**DOI:** 10.1016/j.celrep.2024.115169

**Published:** 2025-01-09

**Authors:** Olivia Lombardi, Ran Li, Faiz Jabbar, Hannah Evans, Silvia Halim, Joanna D.C.C. Lima, Lisa Browning, Helen M. Byrne, Hani Choudhry, Peter J. Ratcliffe, David R. Mole

**Affiliations:** 1 NDM Research Building, University of Oxford, Old Road Campus, Headington, Oxford OX3 7FZ, UK; 2 Department of Cellular Pathology, Oxford University Hospitals NHS Foundation Trust, Headington, Oxford OX3 9DU, UK; 3 Wolfson Centre for Mathematical Biology, Mathematical Institute, University of Oxford, Oxford OX2 6GG, UK; 4 Ludwig Institute for Cancer Research, University of Oxford, Old Road Campus, Headington, Oxford OX3 7DQ, UK; 5 Department of Biochemistry, Faculty of Science, Center of Innovation in Personalized Medicine, King Fahd Center for Medical Research, King Abdulaziz University, Jeddah 3270, Saudi Arabia; 6 The Francis Crick Institute, 1 Midland Road, London NW1 1AT, UK

## Abstract

Clear cell kidney cancers are characterized both by conserved oncogenic driver events and by marked intratumor genetic and phenotypic heterogeneity, which help drive tumor progression, metastasis, and resistance to therapy. How these are reflected in transcriptional programs within the cancer and stromal cell components remains an important question with the potential to drive novel therapeutic approaches to treating cancer. To better understand these programs, we perform single-cell transcriptomics on 75 multi-regional biopsies from kidney tumors and normal kidney. We identify conserved patterns of transcriptional dysregulation and their upstream regulators within the tumor and associated vasculature. We describe recurrent sub-clonal transcriptional consequences of Chr14q loss linked to metastatic potential. We identify prognostically significant conserved patterns of intratumor transcriptional heterogeneity. These reflect co-existing cell states found in both cancer cells and normal kidney cells, indicating that rather than arising from genetic heterogeneity they are a consequence of lineage plasticity.

## Graphical abstract

**Graphical abstract:**
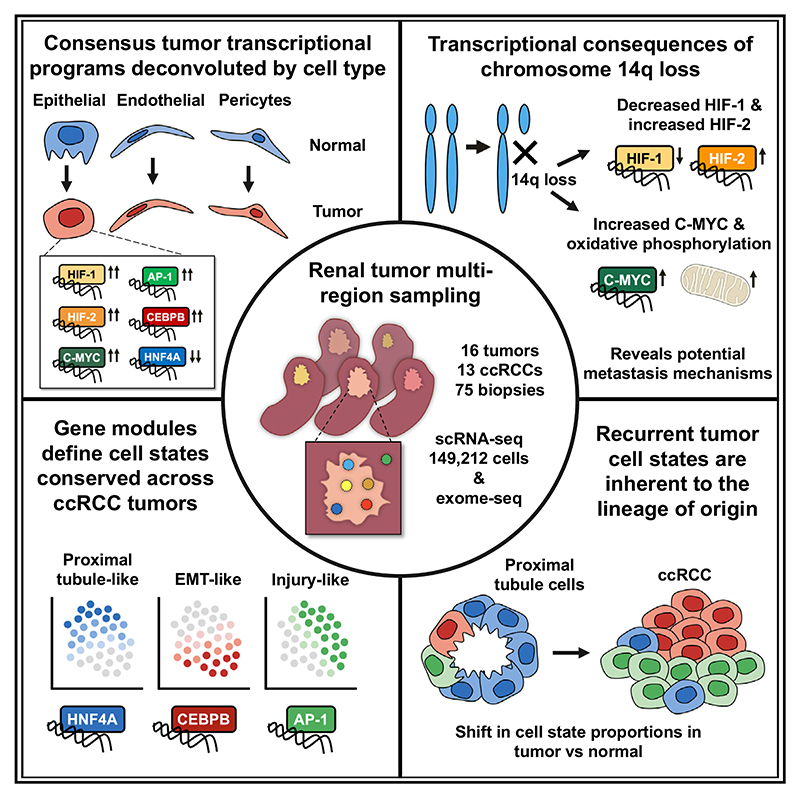

## Introduction

Kidney cancer is among the 10 commonest cancer types with approximately 295,000 new cases and 134,000 deaths worldwide each year.^1^ Clear cell renal cell carcinoma (ccRCC) is the most frequent subtype comprising over 80% of cases.^2^ Tumors are associated with truncal mutation of the von Hippel-Lindau (*VHL*) tumor suppressor gene and constitutive activation of hypoxia-inducible factor (HIF)-driven transcriptional pathways.^3^ Both HIF-1 and HIF-2 isoforms are stabilized and have overlapping, but distinct, roles in ccRCC tumorigenesis.^4^ Additional mutations, affecting the epigenetic regulators polybromo 1 (PBRM1), SET domain containing 2 (SETD2), and BRCA1-associated deubiquitinase 1 (BAP1), often co-occur with VHL inactivation.^5–9^ These may be associated with changes in chromatin accessibility, the HIF pathway, and immune/angiogenic pathways. Additionally, copy-number alterations are common, including Chr9p and Chr14q loss, which are both associated with metastasis.^10^ However, these large-scale chromosomal alterations encompass hundreds of genes and are technically challenging to model *in vitro*, hindering elucidation of their downstream molecular effects.

Two distinguishing hallmarks of ccRCC are marked intratumor heterogeneity and cancer-stromal interactions. Importantly, current therapeutic options for treating advanced kidney cancer largely target these interactions.^11^ Specifically, belzutifan (an HIF-2 inhibitor) and tyrosine kinase inhibitors block the secretion and effects of vascular growth factors on their endothelial cell receptors, and immune checkpoint inhibitors alter interactions with immune effectors.

Intratumor heterogeneity helps drive tumor progression, metastasis, and resistance to therapy.^12–15^ Indeed, ccRCCs exhibit a high degree of subclonal genetic and chromosomal diversity that correlate with clinical outcomes.^10,16^ Extensive intratumor heterogeneity is also observed histologically.^17^ However, the transcriptional consequences of genetic heterogeneity, the transcriptional drivers of morphological diversity, and the role of epigenetic modifications and tumor microenvironment remain unclear.^18^ Over time, these heterogeneous transcriptional programs may allow cellular subpopulations to outcompete other cells, generating accelerated tumor growth or resistance to therapy. Therefore, it is important to understand both the crosstalk between different cell types within the tumor and the transcriptional programs underlying heterogeneous cancer cell subpopulations. Unfortunately, bulk RNA sequencing approaches, by averaging across all cells, are unable to distinguish the transcriptional programs of individual cell types or subclones.

We have undertaken multi-region single-cell RNA sequencing (scRNA-seq) of kidney cancers and surrounding normal renal tissue. This identified conserved programs of transcriptional dysregulation in malignant cancer cells driven by activation of specific transcription factors. These signal to stromal cells within the tumor leading to conserved patterns of transcriptional dysregulation and cellular crosstalk. Combining scRNA-seq data with whole exome sequencing and bulk RNA-seq, we characterized the transcriptional effects of Chr14q loss, a driver of ccRCC metastasis. Analysis of transcriptional heterogeneity within the malignant cancer cells, agnostic to genetics, identified three gene modules representing conserved cell states found in all ccRCC tumors. These comprise two mutually exclusive states (differentiated proximal-tubule-like, and epithelial-mesenchymal transition [EMT]-like states) that exist in a continuum and an uncoupled cell-injury-like state. Each module reflects cell states also observed in the cancer-founder cell type (normal proximal tubular cells) indicating that they reflect non-genetic heterogeneity and cellular plasticity inherent to the lineage of origin.

## Results

### Multi-region tumor sampling and single-cell transcriptomics

**Figure 1:**
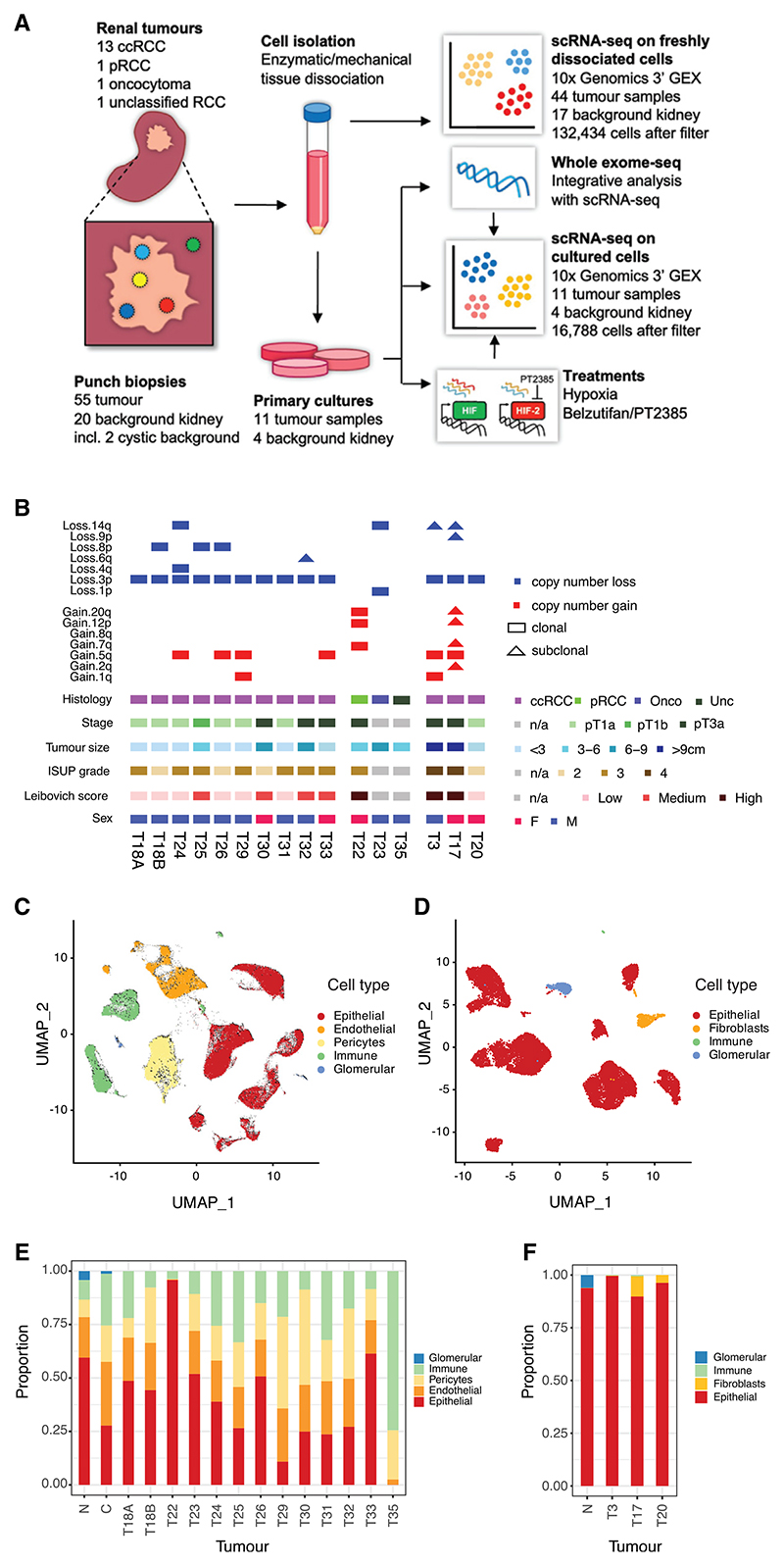
Study overview (A) Schematic of sample analysis. (B) Summary of tumors analyzed, showing CNV (inferred from scRNA-seq data), histological subtype, tumor stage, tumor size, International Society of Urological Pathology (ISUP) grade, Leibovich score, and patient sex. (C and D) Uniform manifold approximation and projection (UMAP) plots of cells from (C) freshly biopsied tumors and (D) primary tumor cultures showing major cell types. (E and F) Stacked bar charts showing major cell type proportions for cells from (E) freshly biopsied tumors and (F) primary tumor cultures. See also Figures S1 and S2.

Multi-region scRNA-seq of primary kidney tumors removed at nephrectomy was performed, focusing on ccRCC (Figure 1A). 55 samples were obtained from 16 tumors in 15 patients (median 3 samples per tumor, range 1–8) (Figures 1B and Table S1). Thirteen were clear cell (ccRCC), one (T22) was papillary (pRCC), one (T23) was renal oncocytoma (oRCC), and one (T35) was unclassified (uRCC). All patients were treatment naive with the exception of patient 35, who was on first-line systemic immunotherapy with nivolumab. Patient 26 had VHL syndrome with an R167W germline *VHL* mutation. 20 background kidney samples were also obtained from macroscopically “normal” kidney tissue within each nephrectomy specimen (range 1–4). Samples from 12 patients (Pt18/22/23/24/25/26/29/30/31/32/33/35) were analyzed immediately following tissue dissociation (biopsy samples). Samples from 3 patients (Pt3/17/20), and normal kidney from Pt18 were used to generate primary cultures prior to separate scRNA-seq analysis.

After filtering, data were obtained from 149,212 cells (132,424 from biopsy samples and 16,788 from cultured samples, Tables S2 and S3). The median number of detected features per cell was 1,778. Hierarchical clustering was performed, and marker genes for each cluster were used to assign broad cell types (Figures 1C, 1D, S1, and S2).^19–22^ Between-patient cell-mixing scores, stratified by cell type, were low for tumor epithelial cells but, in contrast, were high for stromal tumor cells and for normal samples processed in parallel (Figures S1I and S1J). This signifies inter-tumor heterogeneity in tumor epithelial cells in the absence of systemic batch effects affecting other cell types.

Tumor samples had fewer epithelial cells and more pericytes and immune cells than normal samples (Figure S1K), while epithelial cells were the most abundant (median 38.9%, Figure 1E). Tumor samples from Pt35 (on immunotherapy), yielding only 4 epithelial cells, and normal samples from Pt26 (VHL syndrome) containing visible cysts were excluded from further analysis. Following culture, epithelial cells accounted for a greater proportion (median 96.3%) of cells (Figure 1F) likely due to the growth medium selectively promoting growth of these cells. However, these cultures provided material for confirmatory whole exome sequencing and to study the effects of hypoxia/HIF-2 inhibition (Figures S2I–S2P).

Genetic characterization of tumor samples was undertaken using InferCNV (https://github.com/broadinstitute/inferCNV) and Numbat^23^ (Figure 1B) focusing on previously described recurrent renal cell carcinoma (RCC) “driver” events.^16^ All ccRCC epithelial cell clusters exhibited loss of heterozygosity of Chr3p encompassing the VHL gene locus. Tumors T18A and T18B, from patient 18, showed distinct patterns of copy-number variation (CNV), indicating they were separate synchronous tumors. pRCC and oRCC epithelial cells exhibited loss of heterozygosity characteristic of these cancer subtypes^24^ (chr7/17 gain and chr1/14 loss, respectively).

### Conserved transcriptional program dysregulation in cancer epithelial cells

**Figure 2:**
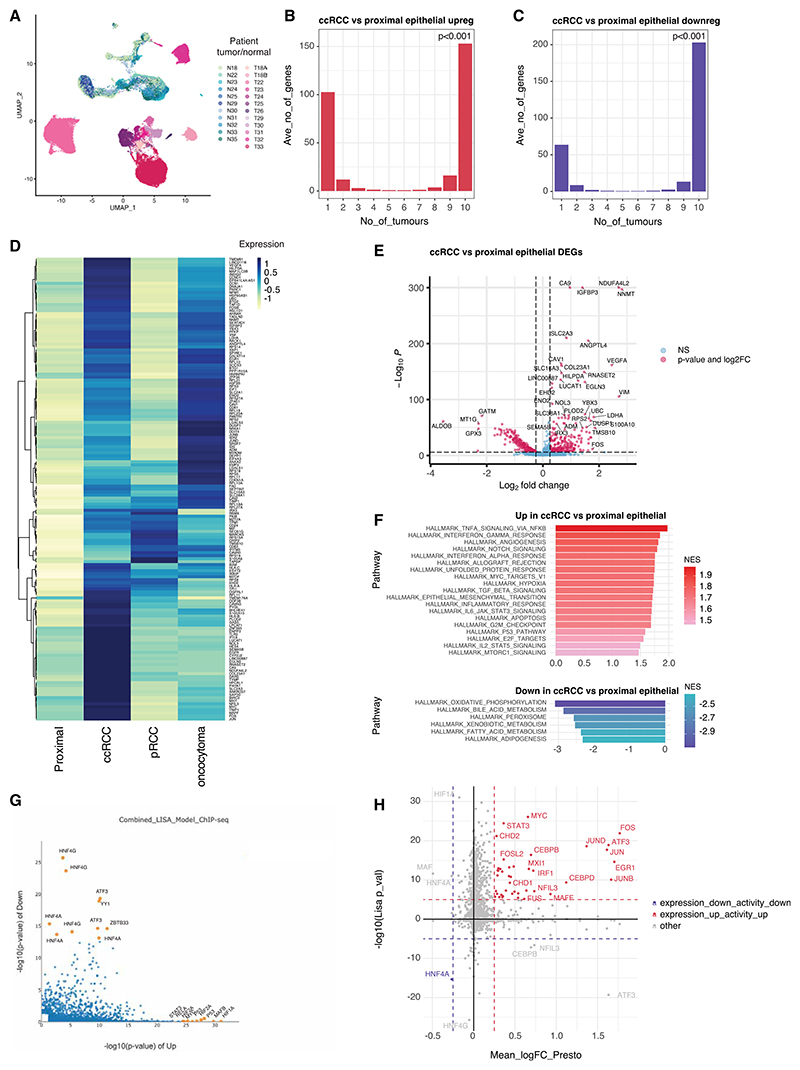
Analysis of conserved patterns of gene dysregulation in epithelial cells from ccRCC samples compared to proximal tubular cells from surrounding normal kidney samples (A) UMAP plot of epithelial cells (freshly biopsied samples) showing tumor/normal samples for each patient (multiple normal or tumor regions combined). (B) Intersection analysis showing overlap between genes upregulated in epithelial cells from each of the 10 freshly biopsied ccRCC tumors compared to normal proximal tubular cells. (C) The same analysis for downregulated genes. In both cases the number of dysregulated genes common to all 10 tumors was significantly higher than expected (*p* < 0.001, by bootstrapping). (D) Heatmap showing expression of the same 153 individual genes in different cell types. (E) Volcano plot showing average log2(fold change) versus average –log10(*p* value) for each gene in epithelial cells from the 10 ccRCCs compared to normal proximal tubular cells. Average expression was calculated for each tumor and then averaged across the 10 tumors, so each tumor was weighted equally. Red denotes genes with log2(fold change) >0.25 or <–0.25 and Bonferroni-adjusted *p* value <0.05. (F) GSEA enrichment of hallmark pathways among genes up- or downregulated in epithelial cells from the 10 ccRCCs compared to normal PTs. (G) Scatterplot showing –log10(*p* value) from LISA analysis of transcription factor binding enrichment at up- vs. downregulated genes in epithelial cells from the 10 ccRCCs compared to normal PTs. (H) Scatterplot showing –log10(*p* value) from LISA analysis of transcription factor binding enrichment at genes up- (positive values) or downregulated (negative transformed values) plotted against average log2(fold change) in mRNA level for the given transcription factor. See also Figure S3 and S4.

We first sought to explore dysregulated gene expression programs in ccRCC cancer epithelial cells relative to normal renal tubular epithelial cells. Epithelial cells from the tumor and normal biopsy samples were reclustered and remapped (Figure 2A). Marker gene analysis of epithelial cell clusters identified a range of expected tubular and glomerular epithelial cell types in normal samples^19,20,25,26^ (Figures S3A–S3I). Normal epithelial cells from different patients clustered closely together with high between-patient cell-mixing scores (Figure S1I). Epithelial cell clusters from tumor samples were distinguished by Chr3p loss (Figure S3J) and high levels of the HIF target and ccRCC marker gene, carbonic anhydrase 9 (CA9)^27^ (Figure S3A).

Notably, normal and cancer epithelial cells clustered distinctly from one another, demonstrating a profound transcriptional change associated with malignant transformation (Figure 2A). However, while normal epithelial cells from different patients overlapped considerably, the malignant epithelial cell clusters demonstrated much less overlap and low between-patient cell-mixing scores (Figures S1I and S1J). This, together with distinct clustering of T18A and T18B (separate tumors from the same patient), indicates inter-tumor heterogeneity, due to true biological variability.

We next examined for conserved patterns of gene dysregulation in tumor epithelial cells compared to normal tubular cells. First, epithelial dysregulated genes in each of the 10 freshly biopsied tumors were separately identified using the Wilcoxon ranksum test. Proximal tubular cells, the most likely cell of origin for ccRCC^20,28^ were used as the normal reference. 3,264 genes were upregulated and 2,337 downregulated in at least one of the 10 ccRCCs. Gene set intersection analysis revealed that deregulated genes were statistically much more likely (*p* < 0.001) to be either unique to an individual tumor or conserved across all 10 ccRCCs (Figures 2B and 2C) and identified conserved gene sets comprising 153 upregulated and 203 downregulated genes (Table S4). Comparatively few genes were upregulated/downregulated in subsets of ccRCC indicating an absence of common ccRCC subtypes defined by large programs of dysregulated genes in cancer epithelial cells. However, subsets of these genes were deregulated in the pRCC and/or oRCC (Figure 2D). Analysis of conserved genes in The Cancer Genome Atlas (TCGA-KIRC) bulk RNA-seq database confirmed upregulation/downregulation in ccRCC tissue samples compared to normal kidney samples and high expression of the 153 upregulated genes in ccRCC tumors relative to other tumor types (Figures S4A–S4C).

Genes upregulated in cancer cells from all 10 ccRCC tumors included the classical HIF target genes *CA9* and *VEGFA*, as well as the atypical mitochondrial subunit *NDUFA4L2*^20^ (Figure 2E). Gene set enrichment analysis (GSEA) using hallmark gene sets (The Molecular Signatures Database [MSigDB]) revealed positive enrichment of hypoxia-associated genes in ccRCC cancer cells (Figure 2F). Additional enriched pathways included tumor necrosis factor alpha (TNF-α)/nuclear factor κB (NF-κB), interferon, angiogenesis, and Notch signaling. Negatively enriched (downregulated) pathways included oxidative phosphorylation, peroxisome, and fatty acid metabolism. Since proximal tubule (PT) cells rely on fatty acid oxidation metabolism via peroxisomes and mitochondria^29^ this is consistent with loss of physiological cellular function in tumors.

We next identified the transcription factors underlying these dysregulated gene programs using epigenetic landscape *in silico* deletion analysis (LISA).^30^ HIF-1α, endothelial PAS domain protein 1 (HIF-2α/EPAS1), and aryl hydrocarbon receptor nuclear translocator (HIF-1β/ARNT) binding were enriched at upregulated genes consistent with their known stabilization in ccRCC (Figure 2G). MAF BZIP transcription factor B (MAFB), P53, V-Rel avian reticuloendotheliosis viral oncogene homolog A (RELA), and signal transducer and activator of transcription 3 (STAT3) binding were also highly enriched. MAFB is a bZIP transcription factor important for podocyte differentiation during development and protects renal tubules from apoptosis.^31^ Consistent with previous bulk studies^32,33^ activities of hepatocyte nuclear factor 4 (HNF4) isoforms, which have roles in PT development/differentiation^34–36^ are depleted, suggesting cellular dedifferentiation.

While HIF regulation is predominantly post-translational, other transcription factors may be regulated transcriptionally. Cross-referencing our LISA analysis with expression analysis identified transcription factors for which both expression and activity were dysregulated in all 10 ccRCCs (Figures 2H and Table S5). Activator protein 1 (AP-1) transcription factors (FOS, FOSL2, JUN, JUNB, and JUND), other immediate-early transcription factors (ATF3, EGR1, and MYC), CCAAT enhancer binding proteins (CEBPB and CEBPD), another avian musculoaponeurotic fibrosarcoma (MAF) family transcription factor (MAFF), immune regulatory transcription factors (IRF1, NFIL3, and STAT3), an EMT master regulator (ZEB2), chromatin remodelers (BRD2, CHD1, and CHD2), epigenetic modifiers (EPC1, JMJD1C, JMJD6, KDM6B, KMT2A, and SAP30), Kruppel-like factor (KLF) transcription factors (KLF4 and KLF10), and avian erythroblastosis virus E26 oncogene homolog (ETS) transcription factors (ETS1 and ETS2) showed upregulation of their mRNA and downstream target genes. The only transcription factor whose expression and activity were downregulated in ccRCC was HNF4A, which directly controls genes critical for PT function^37,38^ again suggesting dedifferentiation in ccRCC cancer cells.

### Conserved transcriptional programs in non-epithelial cancer stromal cells

**Figure 3:**
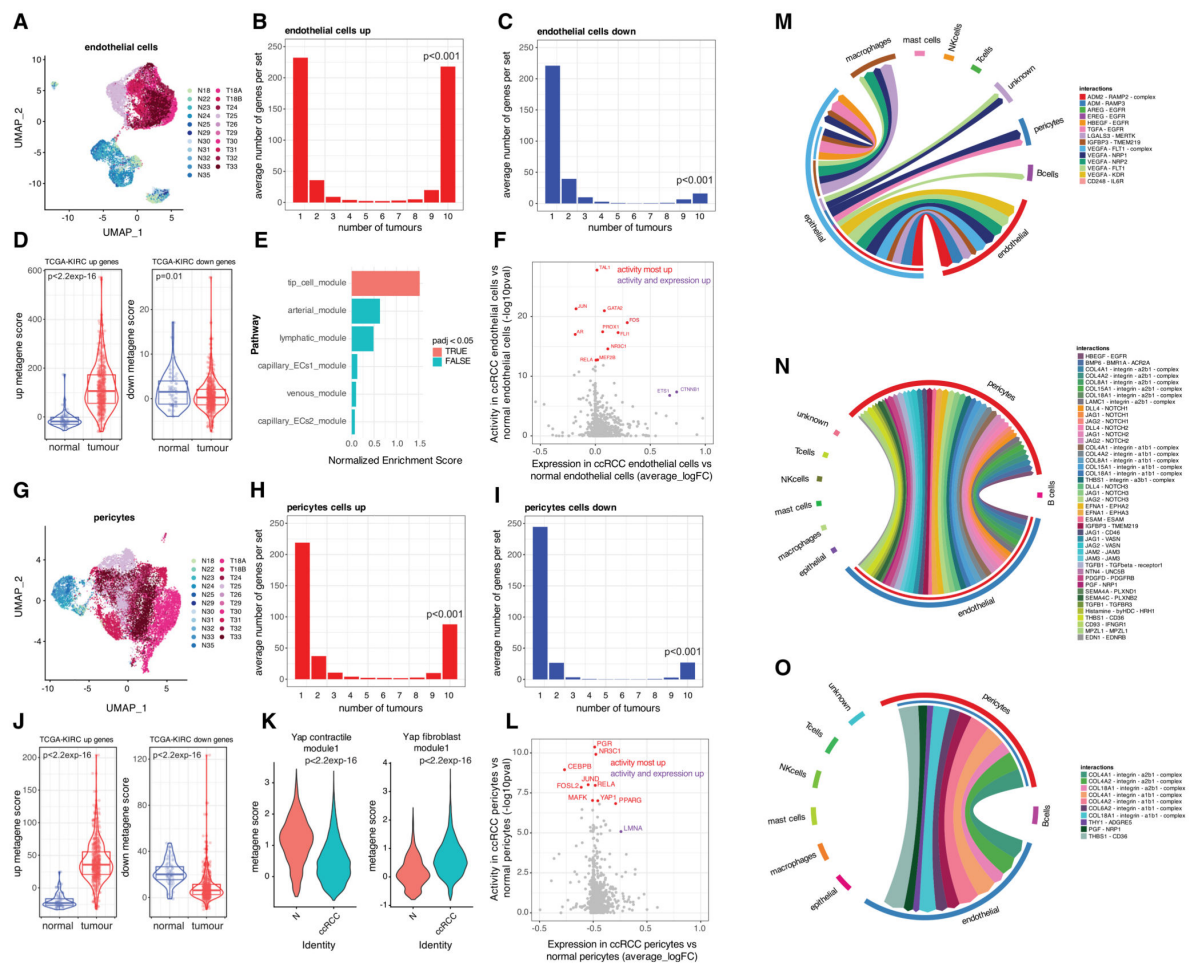
Analysis of conserved patterns of gene dysregulation in endothelial and pericyte cell populations (A) UMAP plot of endothelial cells (freshly biopsied samples) showing sample type and patient. (B) Intersection analysis showing overlap between genes upregulated in endothelial cells from each of the 10 ccRCC tumors compared to normal kidney samples. (C) The same analysis for downregulated genes. In both cases the number of genes dysregulated in all 10 ccRCCs was more than expected (*p* < 0.001 by bootstrapping). (D) Violin and box-and-whisker plots showing composite metagene expression, in bulk RNA analysis of normal and tumor samples from the TCGA-KIRC cohort, for genes up- or downregulated in endothelial cells from all 10 ccRCC compared to normal samples. (E) GSEA showing enrichment of endothelial tip cell marker genes among genes upregulated in endothelial cells. (F) Scatterplot showing –log10(*p* value) from LISA analysis of transcription factor binding at genes upregulated in endothelial cells from all 10 ccRCCs compared to normal samples plotted against the fold change in mRNA for each transcription factor. (G) UMAP plot of pericytes (freshly biopsied samples) showing sample type and patient. (H and I) Intersection analysis to identify genes (H) up- and (I) downregulated in pericytes from all 10 ccRCCs compared to normal samples (*p* < 0.001 by bootstrapping). (J) Violin and box-and-whisker plots showing expression of composite metagenes based on these up- and downregulated genes in bulk RNA-seq analysis of normal and tumor samples from the TCGA-KIRC cohort. (K) Violin plots showing expression of composite metagenes reflecting contractile or fibroblast-like behavior. (L) Scatterplot sowing –log10(*p* value) from LISA analysis versus average –log2(fold change) in mRNA level for transcription factors enriched at genes upregulated in tumor compared to normal pericytes. (M) Chord diagram showing ligand-receptor interactions (identified in CellphoneDB) from ccRCC epithelial cells to other cell types involving genes upregulated in epithelial cells in all 10 ccRCCs. (N) The same analysis for interactions from endothelial cells involving genes upregulated in endothelial cells from all 10 ccRCCs. (O) The same analysis for pericytes. Box-and-whisker plots show median, inter-quartile range, and range. See also Figure S5 and S6.

Beside epithelial cells, tumor and normal biopsy samples contain blood vessels (comprising endothelial cells and pericytes) and immune cells (predominantly macrophages and T cells) (Figure 1G). We examined for conserved programs of gene expression in these non-epithelial stromal tumor cells. Endothelial cells from normal and ccRCC samples clustered separately on uniform manifold approximation and projection (UMAP) plots, suggesting different transcriptional profiles (Figure 3A). Examining genes dysregulated in individual tumors compared to normal samples identified 8,011 genes upregulated and 6,242 genes downregulated in endothelial cells in at least one ccRCC. Gene set intersection analysis (Figures 3B and 3C) revealed that, again, most deregulated genes were unique to individual tumors. However, 218 genes were upregulated and 16 downregulated in endothelial cells in all 10 tumors (Table S6)—significantly more than expected (*p* < 0.001). Both gene signatures were deregulated compared to normal kidney in analysis of bulk RNA-seq data from TCGA-KIRC (Figure 3D). GSEA of endothelial subtype markers^39^ showed enrichment of tip cell markers among ccRCC upregulated genes (Figure 3E). LISA analysis showed enrichment for binding sites of TAL1, GATA2, and FLI, as well as ETS1 and CTNNB1, whose mRNA was also upregulated (Figure 3F). All these transcription factors have been implicated in orchestrating the angiogenic response in endothelial cells.^40–42^

Pericytes from normal and ccRCC samples also cluster separately on UMAP plots (Figure 3G). Gene set intersection analysis again showed statistically significant sets of conserved upregulated/downregulated genes (88/27) in tumor versus normal pericytes (Figures 3H, 3I and Table S7). Metagene signatures derived from these gene sets confirmed upregulation/downregulation in bulk RNA-seq TCGA-KIRC data (Figure 3J). Notably, the atypical mitochondrial subunit NADH dehydrogenase 1 alpha subcomplex subunit 4-like 2 (NDUFA4L2) was upregulated, while NDUFA4 was downregulated (Figures S5A and S5B). While in some settings this switch results from direct transcriptional regulation by HIF^43^ we did not observe HIF activation in ccRCC pericytes (Figure S5C). This suggests that the switch from NDUFA4 to NDUFA4L2 in ccRCC pericytes is due to paracrine signaling from HIF-expressing, ccRCC epithelial cells. We also observed an increase in collagen subunits and genes associated with a previously defined^25^ fibroblast-like vascular smooth muscle cell phenotype together with reduced expression of genes associated with a contractile phenotype indicating a functional change in pericytes associated with tumorigenesis (Figure 3K). Enrichment of binding sites for several transcription factors was also observed (Figure 3L).

Conversely, macrophages and T cells from normal and tumor samples clustered together in UMAP plots (Figures S5D–S5I) and no significant overlap in dysregulated macrophage or T cells genes was observed between each of the 10 ccRCC samples. Thus, our analysis did not distinguish conserved transcriptional profiles in ccRCC macrophages or T cells.

### Analysis of cell-cell interactions in ccRCC tumors

We then examined intratumor cell-cell communication using the CellPhoneDB database^44^ of receptor-ligand interactions. We first analyzed conserved features of cell-cell signaling from ccRCC epithelial cells to other cell types, using the 153 conserved ccRCC upregulated genes (Figure 3M). Direct signaling interactions were observed with several other cell types predominantly between ccRCC epithelial cells and tumor endothelial cells. These involved signaling by the HIF target genes adrenomedullin (ADM) and its paralog adrenomedullin 2 (ADM2) to endothelial receptor activity-modifying protein (RAMP) receptors: galectin 3 (LGALS3) to myeloid epithelial reproductive proto-oncogene tyrosine kinase (MERTK), insulin-like growth factor binding protein 3 (IGFBP3) to transmembrane protein 219 (TMEM219), and vascular endothelial growth factor alpha (VEGFA) to various endothelial receptors (see Figure S6 for additional immunostaining). Direct signaling from ccRCC epithelial cells was also observed to pericytes, macrophages, and B cells as well as cell-type-autonomous signaling back to ccRCC epithelial cells.

Based on the 88 conserved genes upregulated in all 10 ccRCCs, tumor endothelial cells communicated exclusively with tumor pericytes (Figure 3N). Signaling pathways involved included delta-like and jagged canonical notch ligands (DLL4, JAG1, and JAG2) signaling to NOTCH1/2; platelet-derived growth factor D (PDGFD) signaling to its receptor (PDGFRB); binding of collagen (COL4A1, COL4A2, COL8A1, COL15A1, and COL18A1), laminin (LAMC1), and thrombospondin (THBS1) molecules to cell surface integrins; epidermal growth factor (EGF) family members, heparin-binding EGF-like growth factor (HBEGF), and transforming growth factor (TGF)-α; semaphorin 4A and 4C (SEMA4A and SEMA4C); placental growth factor (PGF); netrin 4 (NTN4); and endothelin (EDN1). Communication between ccRCC endothelial cells and pericytes was bidirectional with pericytes expressing high levels of collagens, PGF, THBS1, and Thy-1 cell surface antigen (THY-1) able to interact with cell surface molecules on endothelial cells (Figure 3O).

### Effect of intratumor genetic heterogeneity on transcriptional profile

**Figure 4:**
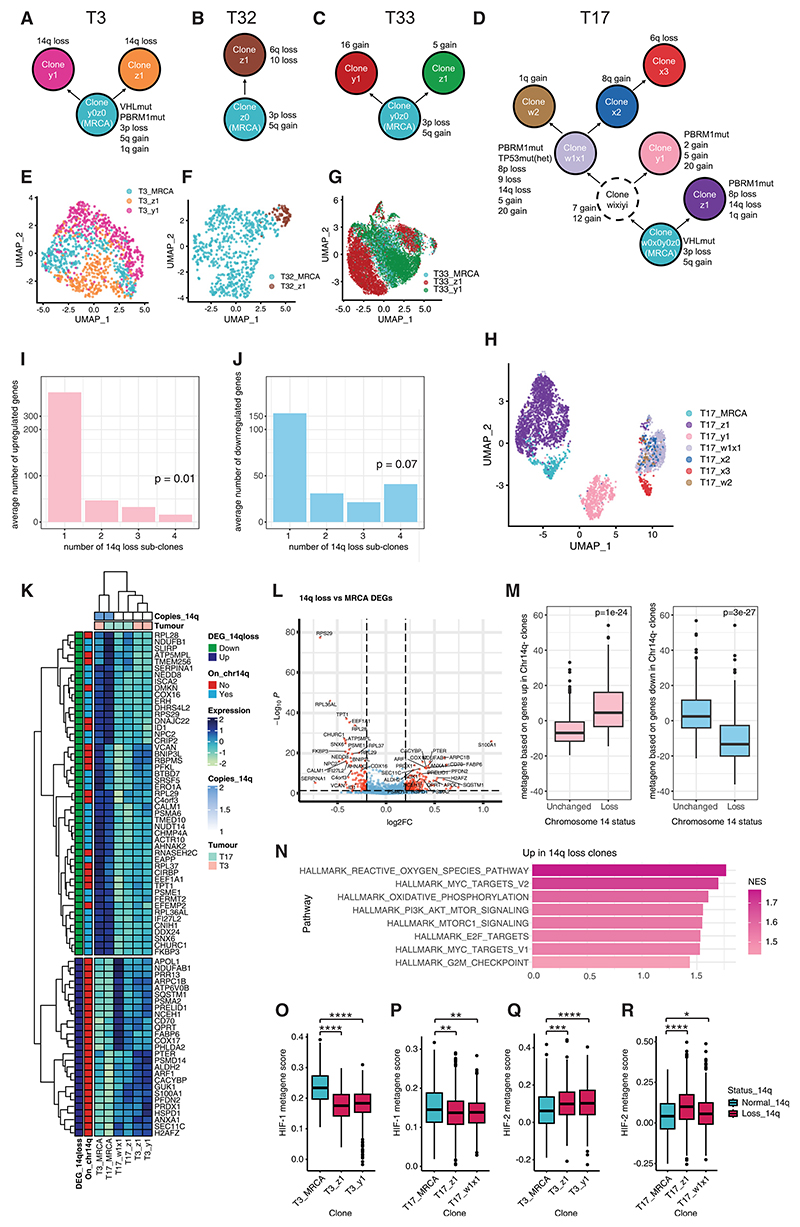
Analysis of intratumor genetic heterogeneity (A–D) Analysis of CNV and loss of heterogeneity (LOH) identified subclonal architecture and tumor phylogenies in epithelial cells from 4 ccRCC tumors. Subclone T17w_i_x_i_y_i_ (D) represents a hypothetical “intermediate” antecedent of T17w_1_x_1_ and T17y_1_. (E–H) UMAP plots of epithelial cells from each of the 4 tumors showing individual subclones. (I and J) Intersection analysis of genes (I) up- or (J) downregulated in the 4 subclones with Chr14q loss compared to their antecedent chr14q wild-type subclones. (K) Heatmap of genes up- or downregulated in all 4 subclones with chr14q loss. (L) Volcano plot showing average log2(fold change) and average –log10(*p* value) for differential gene expression between subclones with chr14q loss and their antecedent chr14q wild-type subclones. Average expression was calculated for each subclone and then averaged across the 4 subclones, so each subclone was weighted equally. Red denotes genes with log2(fold change) >0.25 or <–0.25 and Bonferroni-adjusted *p* value <0.05. (M) Box-and-whisker plot showing expression of composite metagenes, based on genes up- or downregulated in subclones with Chr14q loss, in bulk RNA-seq analysis of tumor samples from the TCGA-KIRC cohort segregated according to Chr14q status. (N) GSEA enrichment of hallmark pathways among genes upregulated in Chr14q loss subclones compared to antecedent 14q wild-type subclones. (O–R) Box-and-whisker plot showing expression of composite metagenes, based on genes up- or downregulated in subclones with Chr14q loss in scRNA-seq analysis of these subclones. *p < 0.05, **p < 0.01, ***p < 0.001, ****p < 0.0001, (Holm-adjusted) Wilcoxon rank-sum test. Box-and-whisker plots show median, inter-quartile range, and range. See also Figures S7–S10.

ccRCC is characterized by high intratumor genetic heterogeneity with recurrent subclonal mutations and chromosomal aberrations. However, it is unclear how these alter the transcriptional output of epithelial cancer cells. We examined the effect of genetic heterogeneity on transcriptional programs in ccRCC. Genetic sub-clones, marked by different patterns of CNV within the epithelial cells, were identified in tumors T3/17/32/33 (Figures S7, S8, and S9), and phylogenetic trees, ordering subclones by evolutionary stage, were constructed (Figures 4A–4H) aided by exome sequencing (exome-seq) of T3/17 (Table S8). Genetic abnormalities often exhibited parallel evolution, occurring independently in multiple subclones, including previously described driver events: *PBRM1* mutation, 14q loss, 1q gain, and 8p loss.^10,16,45,46^

Chr3p loss, a known truncal event in ccRCC,^47^ was consistently observed in all subclones except a T17 subclone, which had copy neutral Chr3p loss of heterozygosity. Chr14q loss, a putative driver of metastasis,^10^ typically encompasses *HIF1A*, which acts as a tumor suppressor in ccRCC.^48–52^ Notably, its paralog, *EPAS1* (HIF2A), which promotes ccRCC tumorigenesis,^48,49,52^ is on Chr2p. However, Chr14q loss encompasses hundreds of genes, and therefore the mechanism associated with tumor metastasis remains unclear. We identified four subclonal incidences of Chr14q loss: two discrete events in T3 and two in T17. Two Chr14q loss subclones had additional CNVs. However, the only common CNV was Chr14q loss. Differential gene expression analysis for each Chr14q loss subclone compared to the most recent common ancestor subclone from the corresponding tumor identified genes dysregulated in all four subclones to delineate the core transcriptional response to Chr14q loss (Figures 4I–4L and Table S9).

49 genes were downregulated in all four subclones. 31 were located on Chr14q and 18 on other chromosomes, distinguishing direct effects of Chr14q loss from secondary effects. 27 genes were upregulated in all four subclones. These included APOL1, one of four genes previously associated with high tumor grade and poor prognosis in ccRCC^53^; SQSTM1 (encoding p62), associated with high-grade ccRCCs and ccRCC oncogenesis^54^; and CD70, implicated in immune evasion.^55^ Composite metagenes based on these gene signatures reflected Chr14q status in bulk RNA-seq analysis of tumors from the TCGA-KIRC cohort (Figure 4M), demonstrating the generalizability of our findings.

Both oxidative phosphorylation and myelocytomatosis oncogene (MYC) targets were significantly enriched among genes upregulated by Chr14q loss, while no hallmark gene sets were enriched among downregulated genes (Figure 4N). Mitochondrial respiration is upregulated in metastatic compared to primary ccRCC, and ccRCC cells with elevated oxidative phosphorylation have increased metastatic potential in mice.^56^ Therefore, the association between Chr14q loss and mitochondrial respiration may, at least partially, explain its association with metastasis. MYC, a proposed oncogene in ccRCC^57^ is a transcriptional target of HIF-2a in ccRCC^58^ and associated with HIF-2-dominant tumors lacking HIF-1.^59^

Despite Chr14q encompassing *HIF1A*, the “hypoxia” hallmark gene set was not significantly enriched among Chr14q loss downregulated genes. However, some genes in the hypoxia gene set were downregulated, while others were upregulated. To test whether HIF-1 and HIF-2 target genes behave differently upon 14q loss, we performed targeted small interfering RNA (siRNA) knockdown of HIF1A/2A in RCC4 cells, followed by RNA-seq analysis to identify HIF-1/2 target genes (Figure S10). Isoform-specific target genes were identified (excluding genes regulated by both isoforms) and leveraged as metagenes to deconvolute HIF-1/2 activity (Tables 10 and S11). Their validity in scRNA-seq analysis was confirmed in primary cultures through their regulation by hypoxia and the HIF-2-specific inhibitor (belzutifan) (Figures S10D and S10E). HIF-1 metagene activity was depleted upon Chr14q loss, consistent with copy-number loss of the *HIF1A* gene (Figures 4O and 4P). Conversely, HIF-2 metagene activity was elevated in response to Chr14q loss (Figures 4Q and 4R). HIF-2 synergizes with MYC^4,59^ and may help drive its increased activity. Together, this indicates that Chr14q loss causes decreased HIF-1 activity, with a reciprocal increase in HIF-2 activity, MYC, and oxidative phosphorylation.

### Conserved patterns of intratumor transcriptional heterogeneity in ccRCC epithelial cancer cells

**Figure 5:**
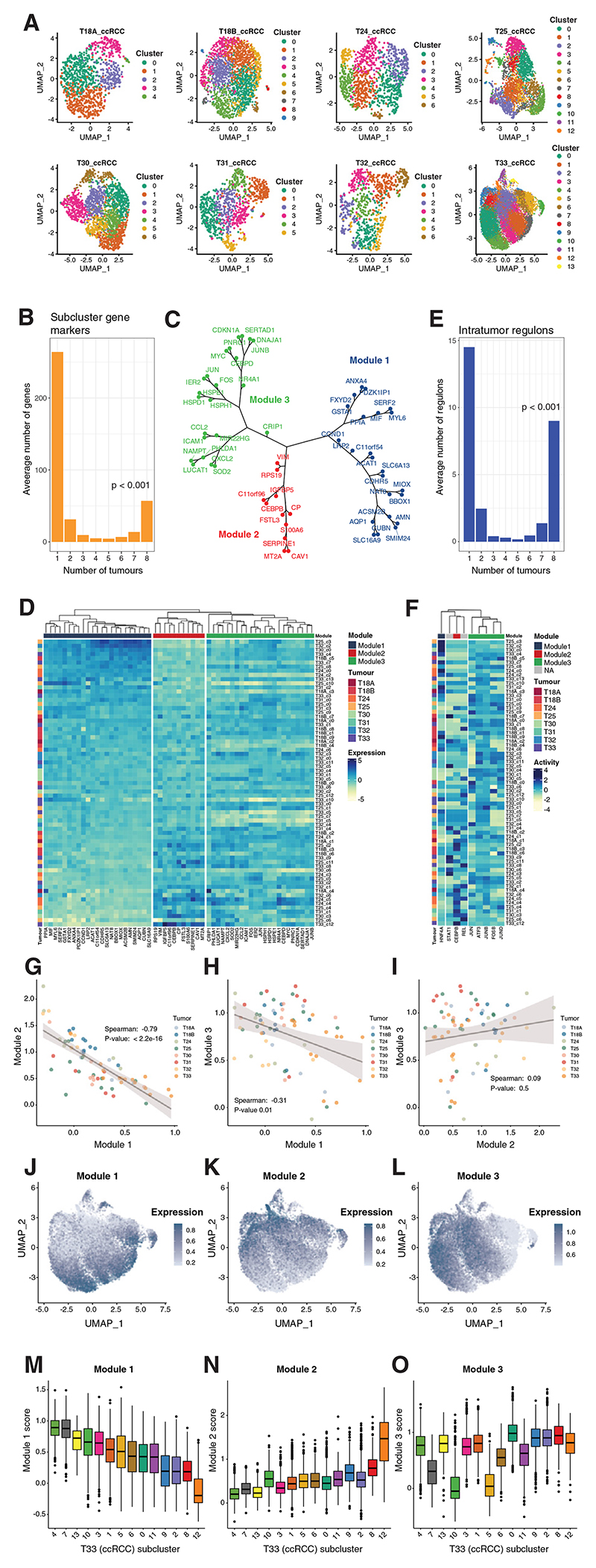
Conserved patterns of transcriptional heterogeneity in epithelial cells from ccRCC samples (A) UMAP plots showing epithelial cells from each ccRCC, remapped and clustered separately. Only tumors with >500 epithelial cells from freshly biopsied samples were analyzed. (B) Intersection analysis of genes marking subclusters in epithelial cells from each of the 8 ccRCCs. Genes marking subclusters in all 8 tumors were enriched (*p* < 0.001 by bootstrapping). (C) k-means hierarchical clustering of cluster markers common to all 8 ccRCC, based on normalized average expression in each cluster, identifying 3 main gene modules. (D) Heatmap showing expression of each common cluster marker gene in individual clusters ordered by the sum of Module-1 gene values. (E) Intersection analysis of transcriptional regulons (defined by SCENIC) exhibiting intratumor heterogeneity in epithelial cells from each of the 8 ccRCCs. Regulons variable within all 8 tumors were enriched (*p* < 0.001 by boot-strapping). (F) Heatmap showing activity of each common regulon in individual clusters ordered by the sum of Module-1 gene values. (G–I) Scatterplots showing mean expression of composite metagenes based on Module-1, -2, and -3 genes in individual tumor clusters. R = Pearson correlation coefficient. (J–L) UMAP plots showing expression of composite metagenes based on Module-1, -2, and -3 genes in epithelial cells from tumor T33. (M–O) Box-and-whisker plots showing T33 cluster-level composite module scores for Module-1, -2, and -3 in clusters ordered by median Module-1 score. Box-and-whisker plots show median, inter-quartile range, and range. See also Figures S11–S14.

We next investigated conserved patterns of intratumor (transcriptional) heterogeneity. Epithelial cells from 8 ccRCCs, with over 500 captured cancer cells, were clustered individually, and “subcluster” markers within each were identified (Figure 5A). Marker genes were more likely to be unique to one tumor or common to all 8 tumors (*p* < 0.001). 57 genes marked cancer cell subpopulations in all 8 ccRCCs (Figure 5B) representing hallmarks of ccRCC intratumor heterogeneity.

Hierarchical clustering of expression of these 57 genes across subclusters in the 8 ccRCCs identified three major groups (modules) of genes (Figures 5C and Table S12). Module-1 genes included PT marker genes (e.g., brush border proteins) determined by cross-referencing markers of normal epithelial cells in our dataset (Table S13). Module-2 genes included several markers of a previously described EMT “meta program”^60^ and ceruloplasmin (CP), previously associated with high ccRCC grade and poor patient prognosis and which may mediate tumor-stroma crosstalk.^9^ Module-3 genes were characteristic of damaged/injured PTs, including immediate-early transcription factors and inflammation markers.^19,61,62^ Immunostaining for representative genes from each module confirmed heterogeneous protein expression (Figure S11).

There was striking negative correlation between expression of Module-1 and -2 genes (Figures 5D and 5G–5O), suggesting that they represent mutually exclusive cellular properties, similar to the findings of a previous study, which noted a negative correlation between PT- and EMT-meta programs.^60^ Expression of Module-3 genes varied on a different axis in UMAP plots (Figures 5I and 5L), suggesting that it represents a distinct cellular property, uncoupled from Module-1 or -2. Module-1 correlated with previously derived PAX8 and HNF1B gene programs^63^ consistent with this module representing a PT-like state (Figure S12). Module-2 negatively correlated with these modules, while Module-3 showed no significant correlation. However, our modules only partially aligned with PAX8 and HNF1B modules in principal component analysis (PCA) analysis indicating that they are at least partially distinct from these programs.

Expression of each module varied continuously across tumors (Figures 5D and 5G–5O) and was leveraged to identify additional genes that covaried (Spearman rank correlation) with each core module in each tumor (Table S14). GSEA, using gene lists ranked according to these correlations, identified oxidative phosphorylation and fatty acid metabolism (metabolic processes related to normal PT function) as most enriched among Module-1-associated genes (Figure S13A). EMT and angiogenesis were most enriched among Module-2-associated genes. Positively enriched Module-1 pathways were negatively enriched among Module-2-associated genes, and vice versa. Pathways most enriched among Module-3-associated genes were TNF-α signaling via NF-κB, interleukin-6 (IL-6)-JAK-STAT3 signaling, and TGF-β signaling.

Barkley et al. reported recurrent cancer cell subpopulations agnostic to tumor type in pan-cancer scRNA-seq analysis.^18^ Barkley oxidative phosphorylation genes were most enriched among Module-1-associated genes (likely reflecting mitochondrial activity related to PT function) and negatively enriched among Module-2-associated genes (Figure S13B). Barkley metal module genes were most enriched among Module-2-associated genes and negatively enriched among Module-1-associated genes. The Barkley stress module was most enriched among Module-3-associated genes.

We then performed single-cell regulatory network interference clustering (SCENIC) analysis^64^ to identify transcription factors with heterogeneous intratumor activity in each of the 8 tumors (Figure S14). 9 transcription factors common to all 8 ccRCCs were identified (Figure 5E, *p* < 0.001). These included HNF4A (Figure 5F), which is required for PT differentiation^37,38^ and is in the extended list of Module-1-correlated genes, and CEBPB in the core Module-2. Consistently, single-nuclei assay for transposase-accessible chromatin using sequencing (ATAC-seq) showed greater chromatin accessibility at CEBPB motifs in EMT-like ccRCC populations and at HNF4A motifs in epithelial-like populations.^9^ Activities of AP-1 family immediate-early transcription factors (JUN/JUNB also in the core Module-3 and ATF3/FOSB/JUND in the extended list of Module-3-correlated genes) also exhibited intratumor heterogeneity across all 8 tumors. Lastly, activity of the inflammatory transcriptional factors, REL and STAT1, exhibited common intratumor heterogeneity, although regulation is likely post-translational since their mRNAs did not vary. HNF4A activity was highest in subclusters with the highest Module-1 expression, whereas CEBPB activity was highest in subclusters with the highest Module-2 expression and exhibited an inverse correlation between each other (Figure 5F). STAT1 and REL activities also clustered with CEBPB, suggesting that they may be additional transcriptional regulators associated with Module-2.

**Figure 6:**
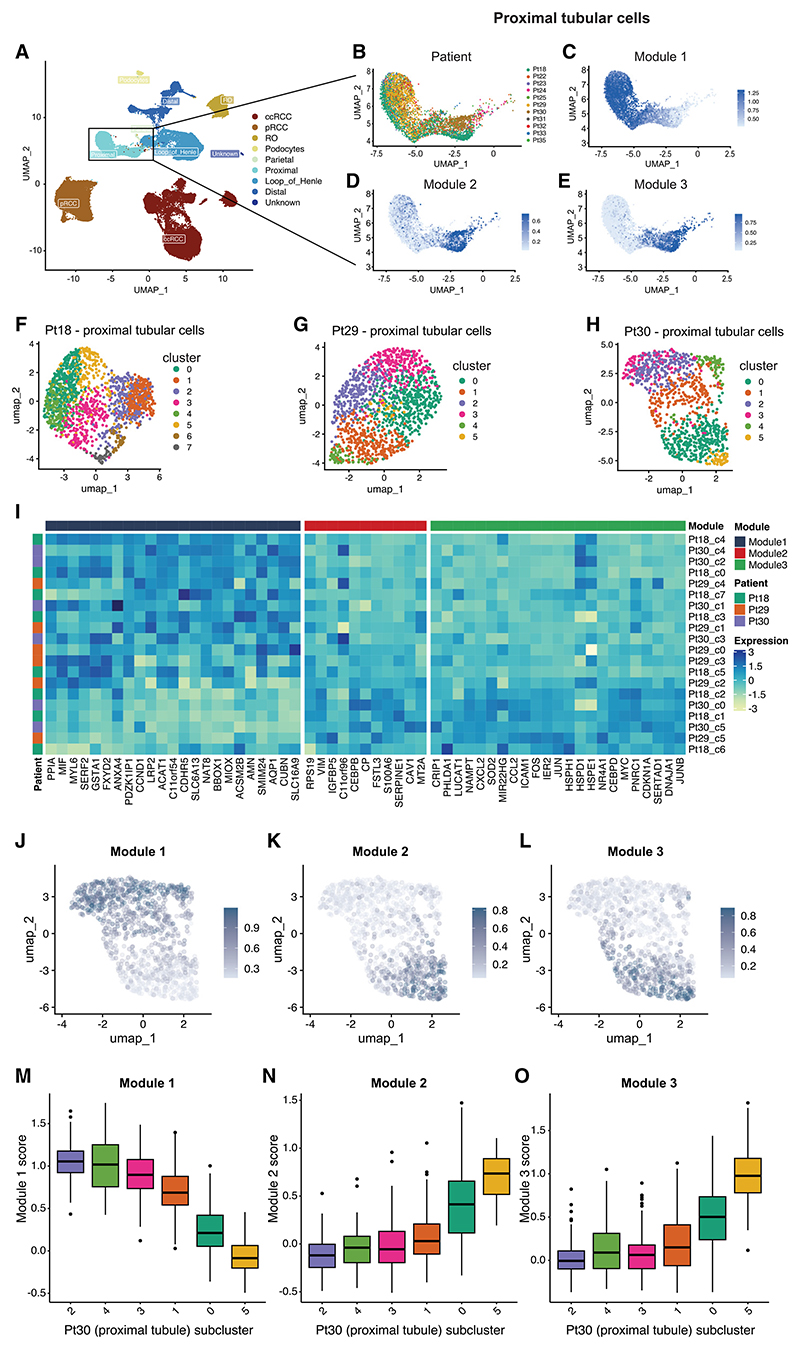
Conserved patterns of epithelial cell heterogeneity are reflected in proximal tubular cells from normal kidney samples (A) UMAP plot of epithelial cells from normal and tumor samples (freshly biopsied) showing cell type. (B) UMAP plot of proximal tubular cells from normal kidney samples showing patient. (C–E) UMAP plots showing expression of composite metagenes based on (C) Module-1, (D) Module-2, and (E) Module-3 genes in proximal tubular cells from normal kidney samples. (F–H) UMAP plots showing proximal tubular cells from normal kidney samples from each patient, remapped and clustered separately. Only patients with >500 PT cells were analyzed. (I) Heatmap showing average cluster-level expression of Module-1, -2, and -3 genes in proximal tubular cells from normal kidney samples with clusters ordered by the sum of Module-1 gene values. (J–L) UMAP plots showing expression of composite metagenes based on Module-1, -2, and -3 genes in normal proximal tubular cells from patient 30 (N30). (M–O) Box-and-whisker plots showing N30 cluster-level composite module scores for modules 1, 2, and 3 in clusters arranged by median Module-1 score. Box- and-whisker plots show median, inter-quartile range, and range.

**Figure 7:**
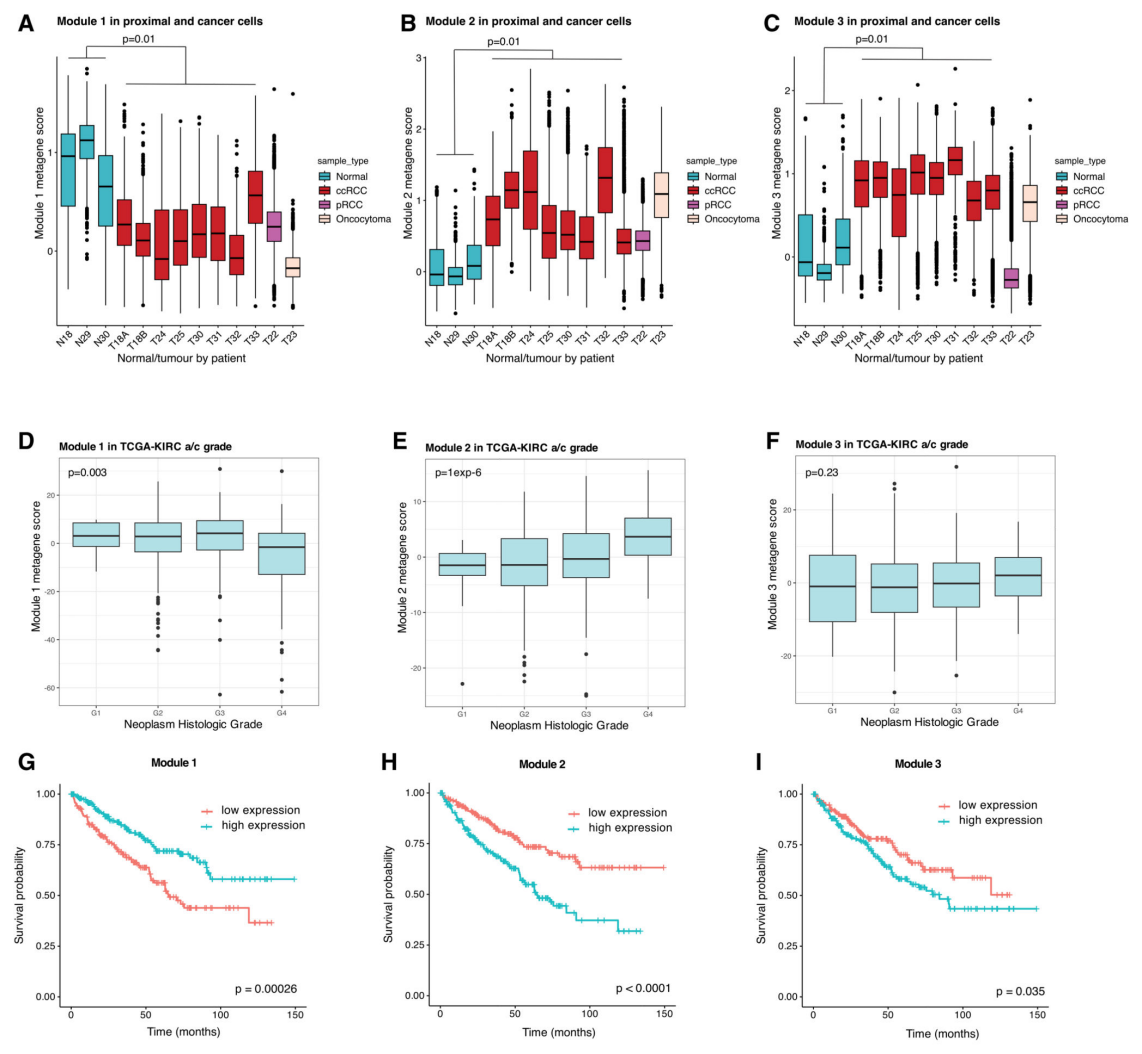
Analysis of Module-1, -2, and -3 gene expression in single-cell and bulk RNA-seq analysis from the TCGA-KIRC cohort Box-and-whisker plots showing expression of composite metagenes based on (A) Module-1, (B) Module-2, and (C) Module-3 in freshly biopsied normal PT cells (blue) and epithelial cells from individual tumors, showing only tumors/patients with >500 cells per group. Box-and-whisker plots showing expression of composite metagenes reflecting (D) Module-1, (E) Module-2, and (F) Module-3 activity in ccRCC tumors (TCGA-KIRC) stratified according to tumor grade (*p* value from Kruskal-Wallis test). Kaplan-Meier plots showing overall survival in TCGA-KIRC patients stratified according to expression of (G) Module-1, (H), Module-2 and (I) Module-3 composite metagenes. Box-and-whisker plots show median, inter-quartile range, and range. See also Figure S15.

Recurrent cell states were identified in every ccRCC tumor irrespective of stage, grade, or size, with modules exhibiting graded expression, suggesting transitional cells with intermediate, dynamic phenotypes. Since Module-1 marks PT differentiation and Module-3 denotes PT injury, we postulated that these cancer cell states might represent lineage plasticity inherent to the cell of origin. We therefore examined expression of each of the modules in normal proximal tubular cells. Expression of all 3 was heterogeneous, varying at both single-cell and cluster level (Figure 6). The existence of these cell states in both normal progenitor cells and in ccRCC cells provides evidence that they are not driven solely by genetic factors in the tumor and indicates cellular plasticity since each tumor will have derived from a single founder cell. Inverse correlation was again observed between modules 1 and 2 (Figures 6C, D, 6I–K, 6M, and 6N). However, modules 2 and 3 were highly correlated (Figures 6D, 6E, 6I, 6K, 6L, 6N, and 6O), indicating tight coupling in normal cells, unlike cancer cells. Although the modules were heterogeneous in both ccRCC and normal PT cells, overall, modules 2/3 had higher and Module-1 had lower expression in cancer cells versus normal cells (Figures 7A–7C). Thus, tumor cells were more likely to adopt EMT/injured-like states underpinned by modules 2/3 and less likely to adopt the PT-like state underpinned by Module-1. Consistent with this, analysis of deregulated transcriptional regulators (Figure 2H) showed downregulation of the Module-1-associated transcriptional regulator (HNF4A) at both the RNA and activity level in ccRCC cells and upregulation of Module-2 (CEBPB) and Module-3 (ATF3, FOS/JUN isoforms)-associated transcriptional regulators.

Module-1 also varied in pRCC, also derived from PT, but not in oRCC, derived from distal tubule (Figure S15). Some variability in modules 2 and 3 was observed in both pRCC and oRCC, suggesting that the associated EMT-like and injury-like cell states are not specific to cancers deriving from the PT lineage. However, while expression of Module-3 is elevated in all 8 ccRCCs, Module-3 expression in the pRCC was comparable to that in the PT (Figure 7C), suggesting that the shift in cell states proportions may be specific to ccRCC.

While the three modules varied within ccRCCs, they also varied between ccRCCs, suggesting different proportions of each cell state (Figures 7A–7C). We leveraged this variation to examine associations with tumor grade and prognosis in the TCGA-KIRC cohort. High Module-1 expression was associated with low tumor grade, while Module-2 increased in higher-grade tumors (Figures 7D–7F). Modules 1 and 2 were prognostic in ccRCC with Module-1 predictive of good and Module-2 predictive of poor prognosis (Figures 7G and 7H).

## Discussion

scRNA-seq allows deconvolution of complex multicellular environments into individual cell types and facilitates examination of transcriptional and genetic heterogeneity within populations of cells. Differential gene expression and gene set intersection analysis identified conserved patterns of transcriptional dysregulation within epithelial ccRCC cells common to all ccRCCs studied. These gene programs are driven by multiple transcription factors in addition to HIF and reflect those modulated in PTs during kidney fibrosis or injury.^19,26,65–67^ This suggests that most ccRCC malignant cells adopt transcriptional programs analogous to injured cell states in the PT epithelium that converge on a dedifferentiated, inflammatory phenotype. Binding sites for many of these transcriptional regulators have previously been reported to overlap with HIF binding sites^68^ suggesting that they may modulate the HIF response.

Similarly, conserved patterns of transcriptional dysregulation were also identified in endothelial cells and pericytes from ccRCC samples. Conversely, we did not identify core transcriptional patterns that distinguished ccRCC macrophages or T cells. This may result from reduced statistical power due to low cell numbers, high stringency of our analysis, or the comparison with immune cells invading the peritumor “normal” kidney, which may resemble those found within the tumors. ccRCC endothelial cells showed upregulation of tip cell marker genes consistent with active sprouting angiogenesis. Pericytes exhibited downregulation of contractile genes with activation of a more fibroblast-like gene profile and a switch from typical to atypical mitochondrial subunit expression. Activation of these genes was greater than in most other cancer types and likely results from paracrine signaling from the tumor epithelial cells involving HIF target genes. Additionally, bidirectional signaling between endothelial and pericyte cell populations was also identified.

ccRCC cancers are characterized by high intratumor genetic heterogeneity, including many putative “driver” events^16,45^ although their transcriptional effects are unclear. We identified tumors with subclonal Chr14q loss (a putative driver of ccRCC metastasis) allowing its transcriptional effects to be studied. Chr14q contains the *HIF1A* gene locus encoding for one of two HIF transcription factors that are constitutively activated in ccRCC. Previous studies indicate a restrictive role for HIF-1 in ccRCC progression, while HIF-2 promotes tumor development.^48,49,51,52,69^ In our study, loss of Chr14q was associated with reduction in HIF-1 activity with concomitant increase in HIF-2 activity. Oxidative phosphorylation, previously shown to be rate-limiting for metastasis in a mouse model^56^ was also upregulated by Chr14q loss. Furthermore, the ccRCC oncogene, SQSTM1, which correlates with both tumor grade and mitochondrial activity in ccRCCs and drives anchorage-independent cell growth^54^ was also upregulated by Chr14q loss.

Our analysis identifies an additional primary source of intratumor transcriptional heterogeneity in ccRCC epithelial cells characterized by three conserved gene programs representing differentiated (Module-1), EMT-like (Module-2), and damaged/injured-like (Module-3) cell states. These gene programs varied continuously across the ccRCC epithelial cell populations with the differentiated and EMT modules representing opposite ends of a continuous spectrum, while the damaged/injured gene module represented an independently varying cellular property. We observed similar heterogeneity in these modules in PT cells from normal kidney, which also resembled previously described modules identified in normal PTs.^19,20,26,28,53,62,65,66,70^ Generally, these separate normal PTs into two main cell states: a well-differentiated state and an injured-like state marked by VCAM1 expression, analogous to our recurrent cancer cell states marked by Module-1 and Module-3, respectively. Although VCAM1 did not meet our stringent criteria to be included in the gene modules due to being variable in 7/8 (rather than 8/8) tumors, it correlated (r = 0.40, *p* = 0.0006) with expression of Module-3 (Figure S12).

The presence of these cell states in both normal and tumor cells suggests that they are not genetically defined. Importantly, in tumors from other tissues, cell states inherent to the corresponding lineage of origin have been described,^18^ implicating this type of heterogeneity as a pervasive feature across tumor biology. Since tumors are derived clonally from single founder cells, it indicates plasticity whereby cells can transdifferentiate between states. Although ccRCC cancer cells have been reported to be most like injured-like VCAM1+ PTs,^20^ the presence of multiple cell states within the tumor cell population makes it difficult to determine the founder state.

The ability of cells to switch between multiple co-existing states has widespread implications for tumor biology, likely contributing to tumor initiation, progression, metastasis, and therapeutic resistance.^71,72^ Specifically, transitioning of tumor cells to and from an EMT-like state has an important role in metastasis. While EMT promotes tumor cell invasion and adherence-independent survival, reversion back to an epithelial-like state once it has reached its destination is also important.^71–73^ Indeed, high EMT gene signature expression has been associated with worse clinical outcomes across many cancers,^74^ and our EMT-like signature correlated with both a higher tumor grade and poorer patient survival. Therefore, perturbing the equilibrium between PT-like (Module-1) and EMT-like (Module-2) cancer cell states represents an opportunity for differentiation therapy. Additionally, our EMT-like module correlated with increased angiogenic signaling, suggesting that EMT-like cells may have heightened sensitivity to antiangiogenic tyrosine kinase inhibitors (TKIs). Future functional studies will be required to understand more precisely how modules 1 and 2 influence cellular phenotype. However, an injury-like signature similar to our Module-3 was previously shown to be transiently induced in tumor thrombi (through which ccRCCs can metastasize) suggesting a role in invasion.^75^

There is also increasing evidence that the presence of multiple cell states within the tumor (and the ability of cells to switch between them) contributes to therapeutic resistance in cancer.^76,77^ While our work has revealed potential drug sensitivities and targets associated with the EMT-like cell state, it is likely that, through plasticity, ccRCC tumors will be able to switch to other cell states, which may be less sensitive to the targeted treatment. Understanding cell state dynamics in ccRCC and the root cause of plasticity will pave the way for novel therapeutic intervention.

### Limitations of the study

By nature, scRNA-seq is a non-spatial analysis of RNA. Further work will be required to study the spatial relationships of our findings at both the RNA and protein level. Genetic analysis of the single-cell data was limited to a few CNVs. New techniques and greater numbers of patients will be needed to study the effects of SNVs. Our methodology enabled a much better recovery of epithelial and blood vessel cells than previous publications, resulting in proportionately fewer immune cells. Our study focused on primary tumors, and extension to tumor metastases will be important.

## Resource Availability

### Lead contact

Requests for further information and resources should be directed to and will be fulfilled by the lead contact, David R. Mole (david.mole@ndm.ox.ac.uk).

### Materials availability

The study did not generate new unique reagents.

## Star★Methods

### Key Resources Table

**Table:** 

REAGENT or RESOURCE	SOURCE	IDENTIFIER
Antibodies		
Purified mouse monoclonal to HIF-1 α	BD Transduction Laboratories	RRID:AB_398272; Cat# 610959
Purified rabbit monoclonal to HIF-2α	Cell Signaling	RRID:AB_10898028; Cat# 7096S
Mouse monoclonal antisera to HIF-2α	In house	190b
Purified rabbit polyclonal to HIF-1 β	Cell Signaling	RRID:AB_10694232; Cat# 5537S
Purified rabbit polyclonal to CA9	Abcam	RRID:AB_2066533; Cat# ab15086
Purified rabbit polyclonal to CA9 (D47G3)	Cell Signaling	RRID:AB_10706355; Cat#5649
Purified rabbit polyclonal to VHL	Cell Signaling	RRID:AB_2716279; Cat# 68547S
Purified mouse monoclonal to β-actin conjugated to HRP	Abcam	RRID:AB_867494; Cat# ab49900
Rabbit Recombinant Monoclonal to Cubilin C-terminal	Abcam	Cat# ab191073
Rabbit monoclonal to Caveolin-1	Abcam	RRID:AB_725987; Cat# ab32577
Anti-ICAM1	Abcam	Cat# ab282575
Rabbit polyclonal to CD31	Abcam	RRID:AB_726362; Cat# ab28364
Rabbit monoclonal to VEGF Receptor 1	Abcam	RRID:AB_778798; Cat# ab32152
TotalSeq-A anti-human Hashtag 1 (GTCAACTCTTTAGCG)	Biolegend	RRID:AB_2750015; Cat# 394601
TotalSeq-A anti-human Hashtag 2 (TGATGGCCTATTGGG)	Biolegend	RRID:AB_2750016; Cat# 394603
TotalSeq-A anti-human Hashtag 3 (TTCCGCCTCTCTTTG)	Biolegend	RRID:AB_2750017; Cat# 394605
TotalSeq-A anti-human Hashtag 4 (AGTAAGTTCAGCGTA)	Biolegend	RRID:AB_2750018; Cat# 394607
TotalSeq-A anti-human Hashtag 5 (AAGTATCGTTTCGCA)	Biolegend	RRID:AB_2750019; Cat# 394609
Biological samples		
Renal tumor and background kidney samples	Oxford Radcliffe Biobank (ORB)	Available on request
Chemicals, peptides, and recombinant proteins		
DMEM/F12	Gibco	Cat# 11320033
Advanced DMEM/F12	Gibco	Cat# 12634010
Dulbecco’s Modified Eagle’s Medium (DMEM)	Sigma Aldrich	Cat# D6429
Glutamax	Gibco	Cat# 35050061
Anti-anti	Gibco	Cat# 15240062
Insulin-transferrin-sodium selenite	Gibco	Cat# 41400045
Triiodo-L-thhryonine	Thermo Fisher	Cat# H34068.MD
Epidermal growth factor	Gibco	Cat# PHG0314
Hydrocortisone	Sigma-Aldrich	Cat# H0888
Hanks balanced salt solution (HBSS)	Gibco	Cat# 24020091
Fetal bovine serum	Sigma-Aldrich	Cat# F7524-500ML
Penicillin-streptomycin	Gibco	Cat# 15140122
Collagenase II	Thermo Fisher	Cat# 17101015
DNase I	Sigma-Aldrich	Cat# 11284932001
Trypsin-EDTA	Sigma-Aldrich	Cat# T4049
Dulbecco’s PBS (DPBS)	Gibco	Cat# 14190144
Bovine Serum Albumin (BSA) Fraction V	Sigma-Aldrich	Cat# 10735086001
DMSO	Sigma-Aldrich	Cat# D2650
PT2385 (Belzutifan)	MedChem Express	Cat# HY-12867
Trypan blue	Gibco	Cat# 15250061
Acridine Orange/Propidium Iodide	Logos Biosystems	Cat# F23001
HIF-1a/HIF1A siRNA	siTOOLS Biotech	Cat# si-K005-3091-HIF1A (Human)
HIF-2a/EPAS1 siRNA	siTOOLS Biotech	Cat# si-G050-2034-EPAS1 (Human)
Control siRNA	siTOOLS Biotech	Part of on-target siRNA pack
RNAiMAX transfection reagent	Thermofisher Scientific	Cat# 13778075
cOmplete protease inhibitor cocktail	Roche	Cat# 11836145001
Critical commercial assays		
MACS Tumor Dissociation Kit (human)	Miltenyi Biotec	Cat# 130-095-929
Nimblegen SeqCap EZ Exome v3	Roche	Discontinued
Twist human core exome	Twist Bioscience	Cat #102027
RNeasy Plus Mini Kit	Qiagen	Cat# 74134
RNase-free DNase Set	Qiagen	Cat# 79254
NEBNext Ultra II Directional RNA Library Prep Kit for Illumina	NEB	Cat# E7765 S/L
DNeasy Blood & Tissue Kit	Qiagen	Cat# 69504
RNase A	Qiagen	Cat# 19101
Chromium Next GEM Single Cell 3’ GEM, Library & Gel Bead Kit v3.1	10x Genomics	Cat# PN-1000121
Chromium Next GEM Chip G Single Cell Kit	10x Genomics	Cat# PN-1000120
Dako Target Retrieval Solution, pH 6	Agilent	Cat# S2369
Dako Peroxidase Blocking solution	Agilent	Cat# S2023
Bovine serum albumin (BSA)	Sigma	Cat# 5482
Dako Envision system	Agilent	Cat# K4003
modified Harris Haematoxylin	Thermo Fisher Scientific	Cat# 72711
DPX mountant	Merck	Cat# 06522
Deposited data		
Single cell RNA-seq analysis of gene expression in fresh and cultured normal and tumor samples	This paper	GEO accession: GSE269819
Bulk RNA-seq analysis of RCC4 cells treated with siRNAs targeting HIF-1alpha and/or HIF-2alpha	This paper	GEO accession: GSE269826
Single cell RNA-seq analysis of gene expression in normoxic/hypoxic primary normal kidney cultures and normoxic ccRCC tumor cultures	Lombardi et al.^78^	GEO accession: GSE200207
Original Western blot images	This paper	Mendeley: https://doi.org/10.17632/7p25n8gwj2.1
Experimental models: Cell lines		
RCC4	Gift from C.H. Buys; validated by detection of the VHL gene mutation (chr3:10,183,841 G > del) in RNA-seq data	RRID: CVCL_0498
Software and algorithms		
Cell Ranger analysis pipeline	10X Genomics	https://www.10xgenomics.com/support/software/cell-ranger/latest
Scrublet	Marine et al.^76^	https://github.com/swolock/scrublet
HTODemux	Stoeckius et al.^79^	https://satijalab.org/seurat/articles/hashing_vignette.html
Seurat (4.0.3)	Hao et al.^80^	https://satijalab.org/seurat/
R (4.0.5)	https://www.r-project.org/foundation/	https://www.r-project.org/foundation/
CellMixS (1.18.0)	https://bioconductor.org/packages/3.18/bioc/html/CellMixS.html	https://bioconductor.org/packages/3.18/bioc/html/CellMixS.html
InferCNV (1.6.0)	InferCNV of the Trinity CTAT Project	https://github.com/broadinstitute/infercnv
Numbat (1.3.2–1)	Gao et al.^23^	https://github.com/kharchenkolab/numbat
CNVkit (0.9.8)	Talevich et al.^81^	https://cnvkit.readthedocs.io/en/stable/
BWA (0.7.15)	Li et al.^82^	https://github.com/lh3/bwa
Picard tools (2.18.20)	http://broadinstitute.github.io/picard/	http://broadinstitute.github.io/picard/
GATK (4.1.9)	Van der Auwera et al.^83^	https://gatk.broadinstitute.org/hc/en-us
gnomAD (4.0)	https://gnomad.broadinstitute.org/downloads	https://gnomad.broadinstitute.org/downloads
SCReadCounts	Prashant et al.^84^	https://horvathlab.github.io/NGS/SCReadCounts/
SAMtools (0.1.19)	Li et al.^85^	http://www.htslib.org/doc/samtools.html
TrimGalore (0.3.3)	https://github.com/FelixKrueger/TrimGalore	https://github.com/FelixKrueger/TrimGalore
HISAT2 (2.05)	Kim et al.^86^	http://daehwankimlab.github.io/hisat2/
HTSeq (0.5.4p3)	Anders et al.^87^	https://htseq.readthedocs.io/en/latest/
DESeq2	Love et al.^88^	https://bioconductor.org/packages/devel/bioc/vignettes/DESeq2/inst/doc/DESeq2.html
UpSetR (1.4.0)	Conway et al.^89^	https://github.com/hms-dbmi/UpSetR
fgsea	Korotkevich et al.^90^	https://github.com/ctlab/fgsea
MsigDB	https://www.gsea-msigdb.org/gsea/msigdb/	https://www.gsea-msigdb.org/gsea/msigdb/
LISA (2.3.0)	Qin et al.^30^	N/A
SCENIC (1.3.1)	Aibar et al.^64^	https://scenic.aertslab.org
CellphoneDB (4.1.0)	Garcia-Alonso et al.^91^	https://github.com/Teichlab/cellphonedb
Ktplots (2.3.0)	https://github.com/zktuong/ktplots	https://github.com/zktuong/ktplots
Other		
*In Vivo2* 400 Hypoxia Workstation	Ruskinn Technology	N/A

### Experimental Model and Study Participant Details

The study was conducted in accordance with the Declaration of Helsinki, and protocols were approved by the Ethics Committee of Oxford University Hospitals NHS Foundation Trust under Oxford Center for Histopathology Research (OCHRe) application numbers 15/A233, 17/A145, 20/A106 and 20/A106b. Specimens were radical or partial nephrectomies collected from individuals undergoing surgery for known or suspected ccRCC (10 males and 5 females – age, ancestry, race and ethnicity not recorded). The RCC4 cell line was authenticated by STR genotyping and exon-sequencing of the VHL mutation and tested for mycoplasma contamination.

### Method Details

#### Tissue sampling

The study was conducted in accordance with the Declaration of Helsinki, and protocols were approved by the Ethics Committee of Oxford University Hospitals NHS Foundation Trust under Oxford Center for Histopathology Research (OCHRe) application numbers 15/A233, 17/A145, 20/A106 and 20/A106b. Specimens were radical or partial nephrectomies collected from individuals undergoing surgery for known or suspected ccRCC.

One or more punch biopsies (4–8 mm) were taken from fresh tumor and background macroscopically normal renal parenchyma as soon as feasible following surgical removal of the kidney and prior to formalin-fixation. Sampled punched out areas of tumor were marked using different colored inks and photographed as a record of sample locations. Where possible samples were taken from macroscopically distinct areas across the tumor, avoiding areas of necrosis. Sampling of tumors did not impact on the clinical diagnostic process.

Samples were annotated by tumor (T)/normal (N)/cystic (C), patient number and, if multiple regions were sampled, by an additional letter (except those from patient 18 who had two separate tumors from each of which one region was taken annotated T18A and T18B). Biopsies were immediately transferred to storage medium (Advanced DMEM/F12 1:1, 1X Glutamax, 1X anti-anti, 1X insulin-transferrin-sodium selenite, 4 ng/mL triiodo-L-thyronine, 100 ng/mL epidermal growth factor, 36 ng/mL hydrocortisone and 20% fetal bovine serum) and put on ice. Biopsies were either processed immediately or kept in the fridge overnight and processed the next morning.

#### Tissue processing

Biopsies were washed twice in cold Hank’s Balanced Salt Solution (HBSS) and minced into ~1mm^3^ cubes. These were incubated, with shaking, for 1 h at 37°C in 7.5mL HBSS containing enzymes from the MACS Tumor Dissociation Kit (37.5μL enzyme A, 300μL enzyme H and 70μL enzyme R) together with collagenase II (193U/ml) and DNase I (3.33 μg/ml). Cells were pelleted by centrifugation at 300*g* for 5 min at 4°C and resuspended in Trypsin-EDTA and incubated in a water bath for 3 min at 37°C. Trypsin was then inactivated by adding 10mL of cold growth medium (Advanced DMEM/F12 1:1, 1X Glutamax, 1X anti-anti, 1X insulin-transferrin-sodium selenite, 4 ng/mL triiodo-L-thyronine, 100 ng/mL epidermal growth factor, 36 ng/mL hydrocortisone and 10% fetal bovine serum). Incompletely dissociated material was allowed to settle, and the suspension was passed sequentially through 100μm, 70μm and 40μm cell strainers. Strained suspensions were repelleted at 300g for 5 min and then incubated in MACS red blood cell lysis buffer for 4 min at room temperature. Ice-cold growth medium was then added and cells repelleted at 300g for 5 min at 4°C. Cell pellets were washed once in 15mL ice-cold Dulbecco’s PBS (DPBS), containing 0.04% bovine serum albumin (BSA), repelleted and finally resuspended in 100μL–500μL of DPBS containing 0.04% BSA to achieve a final concentration of 1000–2000 cells/μL.

#### Primary cultures

Tumor biopsies from Pt3, Pt17 and Pt20 were processed as previously described^78^ to generate cell suspensions for primary cultures. Incompletely dissociated material from the normal kidney biopsy from Pt18 was also plated to derive a culture (N18). Non-adhered cells from all T17 tumor cultures (T17A-G) were pooled to create a ‘T17 mix’ culture, which was used for the DMSO and PT2385 (Belzutifan) treatments. Hypoxic incubations were performed using an *In Vivo*2 400 Hypoxia Workstation (Ruskinn Technology) in an atmosphere containing either 21% (normoxia), 5% (‘physoxia’) or 0.5% (hypoxia) oxygen for 16 h. Primary cultures were maintained in growth medium (Advanced DMEM/F12 1:1, 1X Glutamax, 1X anti-anti, 1X insulin-transferrin-sodium selenite, 4 ng/mL triiodo-L-thyronine, 100 ng/mL epidermal growth factor, 36 ng/mL hydrocortisone and 10% fetal bovine serum) and kept in physoxia (except Pt3 cultures from previously published experiments,^78,92^ which were maintained in normoxia). PT2385 (Belzutifan, HY-12867, MedChem Express) was used at a final concentration of 1μM for 16 h, or 1:1000 DMSO as a vehicle control. Cultures were analyzed at early passage (passages 1–4).

#### Cell hashing for multiplexing primary cultures

Primary cell cultures were detached, made into single cell suspensions, and labeled with TotalSeq-A ‘hashtag’ antibodies as described previously.^78^ Antibodies and associated hashtag sequences were as follows: anti-human 1 (GTCAACTCTTTAGCG), anti-human 2 (TGATGGCCTATTGGG), anti-human 3 (TTCCGCCTCTCTTTG), anti-human 4 (AGTAAGTTCAGCGTA) and anti-human hashtag 5 (AAGTATCGTTTCGCA) (all Biolegend).

#### 10x Genomics GEM generation and library preparation

Cell suspensions were counted (Bio-Rad TC20, Countess II, or LUNA-FL) using Trypan blue or Acridine Orange/Propidium Iodide to assess viability. Approximately 20,000 cells were loaded per channel on the 10x Genomics chip. Single cell 3’ GEX RNA (version 3.1) libraries were prepared according to manufacturer’s instructions (10x Genomics) with single indexing (Pt17, Pt18, Pt20 and Pt22) or dual indexing (Pt23, Pt24, Pt25, Pt26, Pt29, Pt30, Pt31, Pt32, Pt33 and Pt35).

#### Whole exome sequencing

Genomic DNA was prepared using the DNeasy Blood and Tissue kit (Qiagen) and treated with RNase (Qiagen) according to manufacturer’s instructions. DNA integrity was assessed using Genomic DNA Screentape and Reagents on a Tapestation as part of quality control. Whole exome sequencing samples were prepared using the Nimblegen capture kit (Pt3) or the TWIST capture kit (Pt17, Pt18 and Pt20) according to manufacturer instructions.

#### siRNA transfection and bulk RNA-sequencing

RCC4 cells (gift from C.H. Buys) were cultured in high glucose Dulbecco’s Modified Eagle’s Medium (DMEM) (Sigma-Aldrich, D6429) supplemented with 10% fetal bovine serum (FBS) (Sigma-Aldrich F7524) and 1% 100X penicillin-streptomycin antibiotics (Gibco 15140122). Cells were reverse transfected with HIF-1α (HIF1A), HIF-2α (EPAS1), and control siPOOLs (siTOOLS Biotech) using Lipofectamine RNAiMAX Transfection Reagent (Thermofisher Scientific, 13778075) according to manufacturer’s instructions. For transfection, 2x10^5^ cells were seeded on each 6cm dish in 3.5mL of DMEM (Sigma-Aldrich, D6429) containing 10% FBS (without antibiotic) and the final siRNA concentration for each condition was 2nM (control = 2nM controlSi; HIF-1α knockdown = 1nM HIF1Asi + 1nM controlSi; HIF-2α knockdown = 1nM HIF2Asi + 1nM controlSi; double knockdown = 1nM HIF1Asi + 1nM HIF2Asi; although the latter condition was not analyzed for the purpose of this study). After 24h, medium was replaced to fresh antibiotic-free medium. After a further 24h (48h-post transfection), cells were harvested for RNA-seq. RNA-seq was performed as previously described.^78^ Briefly, Total RNA was prepared using the RNeasy Plus Mini kit (Qiagen) and treated with RNase-free DNase Set (Qiagen) according to manufacturer’s instructions. RNA integrity was assessed using RNA Reagents and RNA Screentapes on a Tapestation as part of quality control. PolyA + RNA libraries were then prepared using the NEBNext Ultra II Directional RNA Library Prep Kit for Illumina according to manufacturer’s instructions. All RNA-seq experiments were performed in triplicate in accordance with ENCODE consortium guidelines.^93^

#### Sequencing

Libraries were sequenced on the Illumina NovaSeq 6000 or NextSeq 2000 platforms, according to manufacturer instructions. Paired end sequencing was conducted using the following read configurations: Read 1 = 28bp and Read 2 = 98bp (Pt3, Pt17, Pt18, Pt22, Pt23, Pt24, Pt25, Pt26); Read 1 = 151bp and Read 2 = 151bp (Pt29, Pt30, Pt31, Pt32, Pt33); Read 1 = 28bp and Read 2 = 152bp (Pt35); or Read 1 = 111bp and Read 2 = 111bp (RCC4 bulk RNA-seq).

#### Western blotting

Cell lysates were prepared, and SDS-PAGE/Western blotting was performed as described previously.^78^ Primary antibodies used were anti-HIF-1α (BD cat no. 610959), anti-HIF-2α (in-house 190b or Cell Signaling cat no. 7096S), anti-HIF-1β (Novus Biologicals cat no. NB100-110 or Cell Signaling cat. no. 5537S), anti-CA9 (Abcam cat no. ab15086 or Cell Signaling cat no. 5649S), anti-VHL (Cell Signaling cat no. 68547S) and anti-β-actin (Abcam cat no. ab49900).

#### Immunohistochemistry

Immunohistochemistry was performed as previously described.^70^ Briefly patient 34 FFPE tumor blocks were cut to 4 μm, deparaffinized with xylene and ethanol, and rehydrated with double-distilled water. Sections were subjected to heat-induced epitope retrieval (HIER) using Target Retrieval Solution, pH 6, Dako in a pressure cooker for 20 min. Slides were blocked with Dako Peroxidase Blocking solution (Agilent, S2023) and 5% (w/v) bovine serum albumin (BSA; Sigma, 5482) for 10 and 40 min at room temperature respectively. Antibodies incubation was overnight at 4°C (Anti-Cubilin ab191073, Anti-Caveolin-1 ab32577, Anti-ICAM1 ab282575, Anti-CD31ab28364, Anti-VEGF Receptor 1ab32152) diluted 1:200 each. Signal detection was done using Dako Envision system (Agilent, K4003) with diaminobenzidine (DAB) for 10 min. Slides were counterstained with modified Harris Haematoxylin (Thermo Fisher Scientific, 72711) and a differentiation solution of 0.25% HCL in ethanol for 10 s. Ammonia water solution was used for bluing by immersion for 10 s. Slides were dehydrated and mounted with DPX mountant (Merck, 06522).

### Quantification And Statistical Analysis

#### Processing of scRNA-seq data

Demultiplexing of sequencing results, barcode processing, read alignment, and UMI counting were performed using the Illumina 10x Cell Ranger analysis pipeline v6.1.1 with default parameters. Raw reads for each sample were aligned to GRCh38 reference genome, refdata-gex-GRCh38-2020-A.tar.gz, which was provided by 10X Genomics, using Cell Ranger. Cell hashing was used to pool cultured samples. Cells with detected genes <500, fraction of mitochondrially-encoded reads >0.5, or detected genes >3x median for each sample as well as Scrublet^76^ predicted doublets (expected doublet rate 0.095) were removed. The threshold for mitochondrially-encoded reads was used to allow for the high mitochondrial content in renal tubular epithelial cells and was consistent with an observed bimodal distribution and with previous scRNA-seq analysis of renal tissue. For the pooled, hash tagged, cultured cells, a previously described hashing-based doublet detection strategy, implemented in HTODemux^79^ was employed to identify doublets.

#### Visualization, clustering, and differential gene expression

Downstream analysis was conducted using the R package Seurat v4.^80^ Each gene expression measurement was normalized to total expression in the corresponding cell, multiplied by a scaling factor of 10,000, and log2-transformed. 2,000 variable features were identified based on stabilized variance and scaled for downstream analysis. The original log-normalized expression values were used for all differential expression and gene set level analyzes. Principal components analysis (PCA) was performed using the scaled expression matrix of the 2,000 variable features. The first 30 principal components (PCs) were used for Louvain clustering of cells with a resolution parameter of 1.0. Cell clusters were identified using the FindClusters function within Seurat, which uses a shared nearest neighbor (SNN) modularity optimization based clustering algorithm.^94^ Cell clusters were annotated into broad cell types and epithelial cell sub-types by reference to the expression of previously described marker genes (https://panglaodb.se)^22^ and summarized in Figures 1, S1 and S2. Uniform manifold approximation and projection (UMAP) was performed on the same PCs for visualization in two dimensions. CellMixS (v1.18.0) was used to calculate cell between-patient mixing metrics and to evaluate batch effects.

Differential expression analysis was performed using a two-sided Wilcoxon rank-sum test with Bonferroni FDR correction unless otherwise specified and filtered for an adjusted *p* value < 0.05 and log2-fold change >0.25. Subsets of cells were re-clustered and remapped separately prior to downstream analysis.

#### Inferring copy number variations in scRNA-seq data using InferCNV

Chromosomal CNVs in individual cells were inferred from scRNA-seq data using InferCNV (https://github.com/broadinstitute/inferCNV) with default parameters (cutoff 0.1, cluster_by_groups = T, denoise = T, HMM = T, analysis_mode = “subclusters”). Epithelial cells from normal samples (randomly down sampled to 1,000 cells for the biopsy samples) were used as a reference. Predicted HMM results were then used to assign CNV status to each cell. From InferCNV results, only CNVs found to be recurrent ccRCC driver events in a previous study^16^ were reported.

#### Inferring copy number variations in scRNA-seq data using numbat

Epithelial cells from biopsy tumor samples were further analyzed for CNVs using Numbat (v1.3.2–1), a tool that integrates expression, allele and haplotype information derived from population-based phasing to characterize the CNV landscape in single-cell transcriptomes.^23^ Normal epithelial cells from each patient were used to generate the expression reference and Numbat was applied with default parameters. This analysis confirmed CNVs identified by InferCNV. In addition, a focal copy loss at the telomeric end of 3p loss encompassing the VHL locus was identified in T33, which was not detected by InferCNV. One further subclonal copy loss was identified, using Numbat, in T32 (identifying the T32y_1_ subclone) and two subclonal copy losses were identified in T33 (identifying T33y_1_ and T33z_1_ subclones), which were not detected by InferCNV. For visualization of subclones on UMAP plots, ‘ambiguous’ cells that could not be assigned to a subclone by Numbat (posterior probability <0.95) were excluded. Only CNVs found to be recurrent ccRCC driver events in a previous study^16^ were reported, except when non-driver CNVs were required to distinguish genetic subclones.

#### Somatic copy number analysis of exome-seq data

Exome sequencing was performed on Pt3, Pt17 and Pt20. CNVkit v0.9.8^81^ was used to identify CNVs from paired tumor-normal sequencing data using “--drop-low-coverage” to drop bins with low coverage and “–center mode” to recenter the log2 values. Log2 values were then converted to copy number to show copy losses or gains. For tumor samples with a single or dominant subclone, the CNVkit profile confirmed CNVs found by InferCNV. CNVkit analysis identified additional focal CNVs that were too small to be identified by InferCNV (e.g., losses on 8p or 6q) and demonstrated 5q tetraploidy in some samples.

#### Somatic variant analysis in exome-seq data

Fastq files were mapped to reference genome hg38 using bwa 0.7.15.^82^ Aligned bam files were sorted and duplicated reads marked by picard 2.18.20 (http://broadinstitute.github.io/picard/). bam files were then processed using the GATK 4.1.9 tools BaseRecalibrator and ApplyBQSR (https://gatk.broadinstitute.org/hc/en-us).^83^ Bam files were ordered according to the reference genome using picard. Mutect2 from GATK 4.1.9 was used to call somatic variants in tumor samples, and filtering using gnomAD v4.0 (https://gnomad.broadinstitute.org/downloads). Potential somatic mutations were further filtered by FilterMutectCalls in GATK 4.1.9. The final mutation lists were annotated using Funcotator in GATK 4.1.9 using the data source funcotator_dataSources.v1.7.20200521s. Analysis focused on previously described driver mutations.^16^ Considering the clonal composition of samples (derived from scRNA-seq data) enabled mapping of variants to specific subclones.

#### Allele frequency analysis in exome-seq data

HaplotypeCaller in GATK 4.1.9 was used to call germline variants. Heterozygous germline variants identified in normal samples with a read depth >30 and balanced allele frequency of reference/alternative between 0.35 and 0.65 were leveraged to perform allele frequency analysis in patient-matched tumor samples. This identified mirrored subclonal allelic imbalance (differential loss of maternal and paternal chromosome copies in different subclones) indicating independent occurrences of the same CNVs. Specifically, this analysis identified loss of different 14q copies in samples from T3 (which was not possible using InferCNV) distinguishing T3y_1_ and T3z_1_ subclones from each other. Similarly, gains of different chr5 and chr20 copies were identified in different T17 samples, distinguishing them as events occurring independently in subclones T17y_1_ and T17w_1_x_1_.

#### Allele frequency analysis in scRNA-seq data

SCReadCounts^84^ was used to generate cell-SNV matrices containing the absolute variant- and reference-read counts from barcoded scRNA-seq alignments. Specifically, NM and MD tags were added to scRNA-seq bam files using samtools^85^ calmd -b, and the varLoci function from SCReadCounts used to get potential variants from the normal scRNA-seq samples (minimal alternative reads = 10). Then, scReadCounts was used to generate the reference reads count and alternative reads count from both the normal and tumor scRNA-seq samples for the potential variants generated using varLoci with the parameter -C STARsolo.

To validate the mirrored subclonal allelic imbalance at 14q in T3 subclones (T3y_1_ and T3z_1_) suspected from exome-seq, heterozygous sites from the normal sample (N3) were selected using both exome-seq data and scRNA-seq data. From exome-seq data, sites with at least 10 alternative reads, 10 reference reads and balanced allele frequency (alternative reads frequency between 0.3 and 0.7) were selected. From the scRNA-seq data, cells from each T3 sample (T3A and T3B) were stratified by 14q status, according to inferCNV results. Then, using the reads counts from SCReadCounts, heterozygous sites were selected from N3 scRNA-seq data, requiring that the alleles must overlap with heterozygous sites identified in exome-seq and be balanced in N3 scRNA-seq data (alternative reads frequency between 0.3 and 0.7). Of those sites, those with over 50 reads collectively across T3 samples were selected, yielding 7 high confidence sites. Reference and alternative allele frequency was calculated at these sites in T3 cancer cells stratified by sample and 14q status, as well as N3 cells. This confirmed an allelic bias in cells with 14q loss from different T3 samples, indicating they lost different 14q copies.

This was also applied to investigate a cluster of T17 cells (mapping to clone T17y_1_) in which 3p loss was not detected by InferCNV, despite expressing CA9 and other HIF target genes. To test for copy neutral loss of heterozygosity, heterozygous sites on 3p in the Pt17 normal sample (N17) were selected as above, specifically in the region undergoing copy loss in other ccRCCs (chr3:1-86900000). Sites from exome-seq were selected for those with a minimum 10 reference and 10 alternative reads, and allele frequency between 0.25 and 0.75. From scRNA-seq data, the sites were further selected for those having at least 10 reads from the N17 and T17 samples collectively, and at least 5 reads in the 3p copy neutral T17 cluster under investigation. Additionally, sites taken forward had at least 3 reference and 3 alterative reads in N17 scRNA-seq data. This yielded 9 high-confidence sites, which showed a balanced allele frequency in N17, yet the cluster under investigation only showed signal from one allele. This demonstrated that indeed the T17y_1_ subclone underwent copy neutral loss of heterozygosity at 3p, whereby it originally lost 3p and then duplicated the remaining copy.

#### RNA-seq analysis of RCC4 cells

RNA-seq data was analyzed as previously described.^78^ Briefly, Illumina adaptor sequences were trimmed using TrimGalore (0.3.3). Reads were aligned to Genome Reference Consortium GRCh37 (hg19) using HISAT2 (2.05) (http://daehwankimlab.github.io/hisat2/).^86^ Non-uniquely mapped fragments were excluded using Picard tools (2.0.1) (http://broadinstitute.github.io/picard/). Total read counts for each UCSC-defined gene were extracted using HTSeq (0.5.4p3)^87^ with ‘intersection-strict’ mode, and significantly regulated genes were identified using DESeq2, pairing conditions by biological replicate.^88^

#### Gene set intersection analysis

Gene set intersection analysis was performed using UpSetR (https://upset.app).^89,95^ Significance was determined using a bootstrapping approach with 1,000 iterations.

#### Gene set enrichment analysis

Gene set enrichment analysis (GSEA) was performed using 10,000 permutations, weighted enrichment score and pre-ranking of genes.^90,96,97^ The ranking metric (log_2_ fold change) was first calculated for each tumor/subclone and then averaged across tumors/subclones to avoid biasing the analysis toward tumors/subclones with more cells. Genesets were accessed through MSigDB (https://www.gsea-msigdb.org/gsea/msigdb/) or published studies.^18,39^

#### Transcriptional regulator prediction using LISA

Upstream transcriptional regulators of gene programs were identified using epigenetic Landscape In Silico deletion Analysis (LISA – v2.3.0 - https://github.com/liulab-dfci/lisa2#).^30^ For epithelial cells, tumor (vs. normal) dysregulated transcription regulators were calculated by comparing the consistently upregulated (*n* = 153) and consistently downregulated (*n* = 203) genes in ccRCC cancer cells (vs. proximal tubules). The first sample *p*-value was selected for each transcriptional regulator. For transcriptional regulators enriched in the tumor downregulated (proximal tubule upregulated) genes, the *p*-value was negatively transformed so that it could be correlated with log2FC in expression of the transcriptional regulator. The non-epithelial cell LISA analysis was conducted by inputting the consistently tumor upregulated genes only, since there were insufficient numbers of genes consistently downregulated (<50 genes).

#### Transcriptional regulator prediction using SCENIC

Single-cell regulatory network inference and clustering (SCENIC – v1.3.1 - https://scenic.aertslab.org)^64^ was used to identify transcription regulators showing intratumor heterogeneity between cancer epithelial cells. For SCENIC analysis, variable regulons were calculated for each tumor, and the 9 regulons displaying intratumor heterogeneity in all 8 ccRCCs analyzed were described. Activities of the 9 regulons were mapped to individual cancer epithelial cells (each tumor separately), then scaled and centered across cells from each tumor. The average activity was calculated for each subcluster in each tumor, and then the 8 matrices merged by regulon. Activities for each regulon were scaled across subclusters before being visualized on a heatmap.

#### Cell-cell interaction analysis

Cell-cell interactions were analyzed using CellphoneDB v4.1.0^91^ and visualized using ktplots version 2.3.0 (https://github.com/zktuong/ktplots).

#### TCGA RNA-seq data and prognosis analysis

FPKM-UQ normalized RNA-seq data for 9,760 primary tumor samples and 730 normal samples were obtained from https://portal.gdc.cancer.gov/repository on 07.09.2021 using the gdc-client version 1.5.0 and the following advanced filters: cases.project.program.name in ["TCGA"], files.analysis.workflow_type in ["HTSeq - FPKM-UQ"], files.data_category in ["transcriptome profiling"], files.experimental_strategy in ["RNA-Seq"] and either cases.samples.sample_type in ["primary tumor"] or cases.samples.sample_type in ["solid tissue normal"].

#### Deciphering intratumor expression programs and meta-programs

Tumors with more than 500 captured cancer epithelial cells (8 of 10 ccRCCs) were analyzed for intratumor expression programs. Epithelial cells from each tumor were reclustered and remapped separately (to derive tumor ‘subclusters’), as described above. Small subclusters suggestive of cell doublets (low expression of the ccRCC marker CA9 and high expression of non-epithelial cell markers) or poor-quality cells (e.g., low reads/features) were excluded. The remaining cells were then reclustered and remapped a second time. Subcluster marker genes were then distinguished for each tumor as described above. Analysis of intersecting gene sets identified 57 subcluster marker genes common to all 8 ccRCCs.

The expression of each of these 57 genes was then scaled and centered across cells from each tumor and used to calculate the average expression for each tumor subcluster. Data from the 8 tumors were then merged, and each rescaled across the subclusters. The Euclidean distance was calculated between genes, and hierarchical clustering (complete linkage) performed on the distance matrix to construct a dendrogram tree. The tree was cut into 3 clusters of genes, and the clusters of genes defined as gene modules. Gene module ‘expression’ was calculated for single cells using the Seurat “AddModuleScore” function. Cutting the dendrogram tree into 3, 4 and 5 clusters was tested, but the associated gene modules with 4/5 clusters exhibited high spearman correlation coefficients between certain pairs of modules in tumor subclusters, and therefore 3 clusters was selected.

Leveraging the three gene modules as ‘core modules’, we then performed spearman correlation coefficient rank tests to identify genes that correlated with each module in each tumor across individual cancer epithelial cells. Owing to the large number of cells, tumor T33 was randomly down sampled to 500 cells per sample (2,500 total) for this analysis. The average correlation coefficient across all 8 tumors was used as the gene ranking metric for subsequent GSEA. Positively correlated genes (adjusted *p* < 0.05) common across all 8 tumors defined the ‘extended modules’.

#### Generation of HIF-1 and HIF-2 specific gene metagenes

Genes downregulated in RCC4 cells by HIF1A siRNA or HIF2A (EPAS1) siRNA (Benjamini–Hochberg corrected *p* < 0.1 and a log_2_ fold change <-0.263.), relative to control siRNA, defined HIF-1 and HIF-2 target genes respectively. Non-overlapping HIF-1 and HIF-2 target genes defined the HIF-1 specific and HIF-2 specific metagenes (excluding HIF1A and HIF2A/EPAS1 themselves). HIF-1 and HIF-2-specific metagene ‘expression’ scores were assigned to single cells using the Seurat “AddModuleScore” function with “search = TRUE” to ensure the correct alias was used in the scRNA-seq data.

## Supplementary Material

Supplemental information can be found online at https://doi.org/10.1016/j.celrep.2024.115169.

Table S1

Table S2

Table S3

Table S4

Table S5

Table S6

Table S7

Table S8

Table S9

Table S10

Table S11

Table S12

Table S13

Table S14

Supplemental information

## Data Availability

**Data:** Seurat objects and bulk RNA-seq data are available at Gene Expression Omnibus (GEO): GSE269819 and GSE269826 (patient 3 data were previously uploaded individually to GSE200207). Original western blot images are available at Mendeley (https://doi.org/10.17632/7p25n8gwj2.1). **Code:** This paper does not report original code. **Other items:** Microscopy data reported in this paper will be shared by the lead contact upon request. Any additional information required to reanalyze the data reported in this paper is available from the lead contact upon request.

## Highlights

- ccRCC exhibits hallmark transcriptional programs in cancer and stromal cells
- Conserved cancer-stroma interactions characterize ccRCC tumors
- Chr14qloss is associated with altered HIF-1/2 and C-MYC transcriptional responses
- Cancer cell states recapitulate normal cell subpopulations, reflecting plasticity

## In brief

Lombardi et al. identify conserved dysregulated transcriptional programs within ccRCC and associated vasculature. They describe the transcriptional consequences of metastasis-associated Chr14q loss. They identify additional prognostically significant gene modules reflecting conserved intratumor transcriptional heterogeneity and marking co-existing cell states and lineage plasticity found in both cancer and normal cells.
