# Supplementary material for: Conserved patterns of transcriptional dysregulation, heterogeneity, and cell states in clear cell kidney cancer
Source: Cell Rep. Author manuscript; Available in PMC 2025 Jul 8. (PMC7617885; doi:10.1016/j.celrep.2024.115169)
Supplement: Supplemental information [file EMS206782-supplement-Supplemental_information.pdf]

Cell Reports, Volume *44*

Supplemental information

Conserved patterns of transcriptional dysregulation, heterogeneity, and cell states in clear cell kidney cancer

Olivia Lombardi, Ran Li, Faiz Jabbar, Hannah Evans, Silvia Halim, Joanna D.C.C. Lima, Lisa Browning, Helen M. Byrne, Hani Choudhry, Peter J. Ratcliffe, and David R. Mole

Figure S1

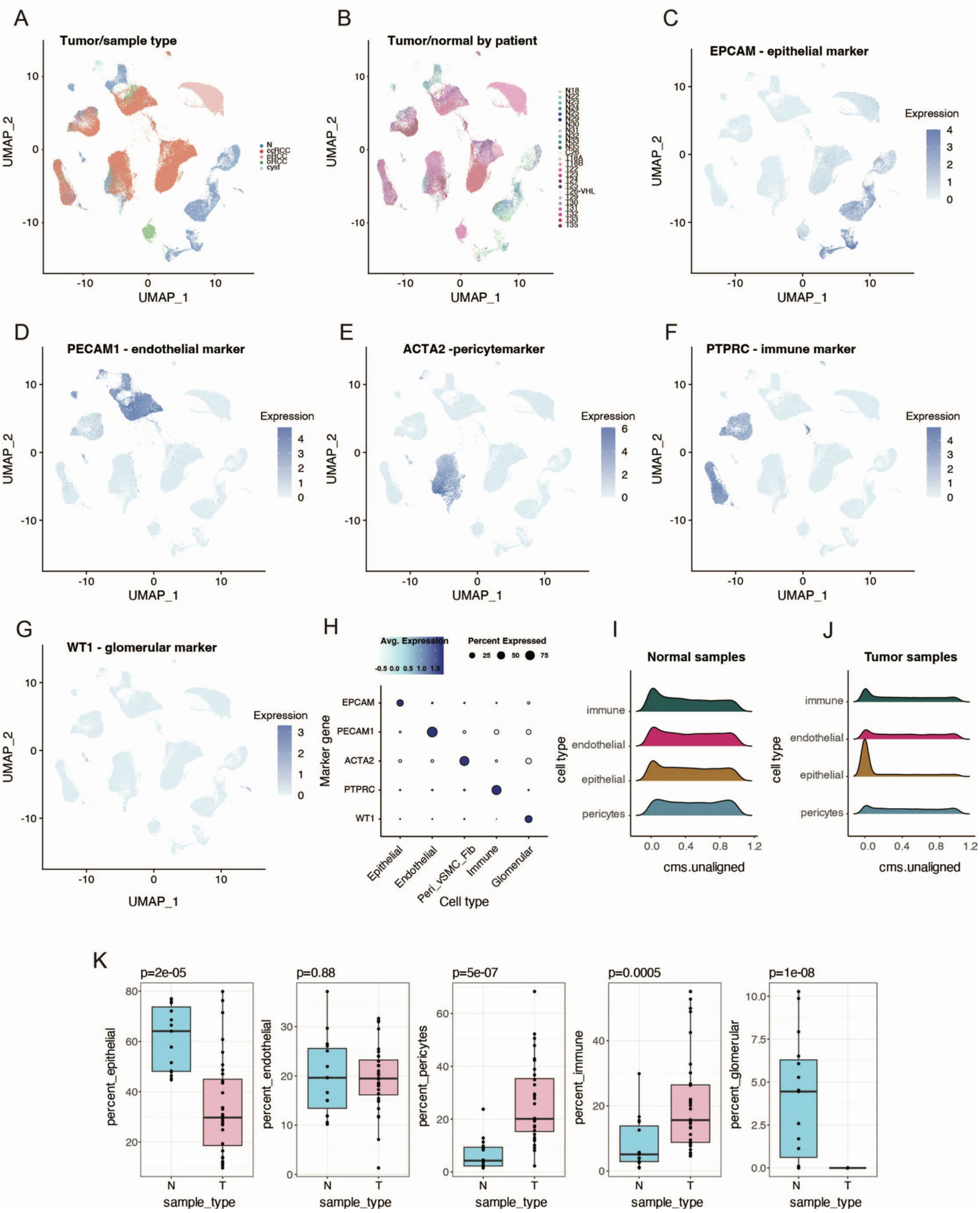
A
Tumor/sample type
B
Tumor/normal by patient
C
EPCAM - epithelial marker
D
PECAM1 - endothelial marker
E
ACTA2 -pericytemarker
F
PTPRC - immune marker
G
WT1 - glomerular marker
H
Avg. Expression
Percent Expressed
Marker gene
Cell type
I
Normal samples
J
Tumor samples
cell type
cms.unaligned
K
p=2e-05
p=0.88
p=5e-07
p=0.0005
p=1e-08
sample_type

Figure S2

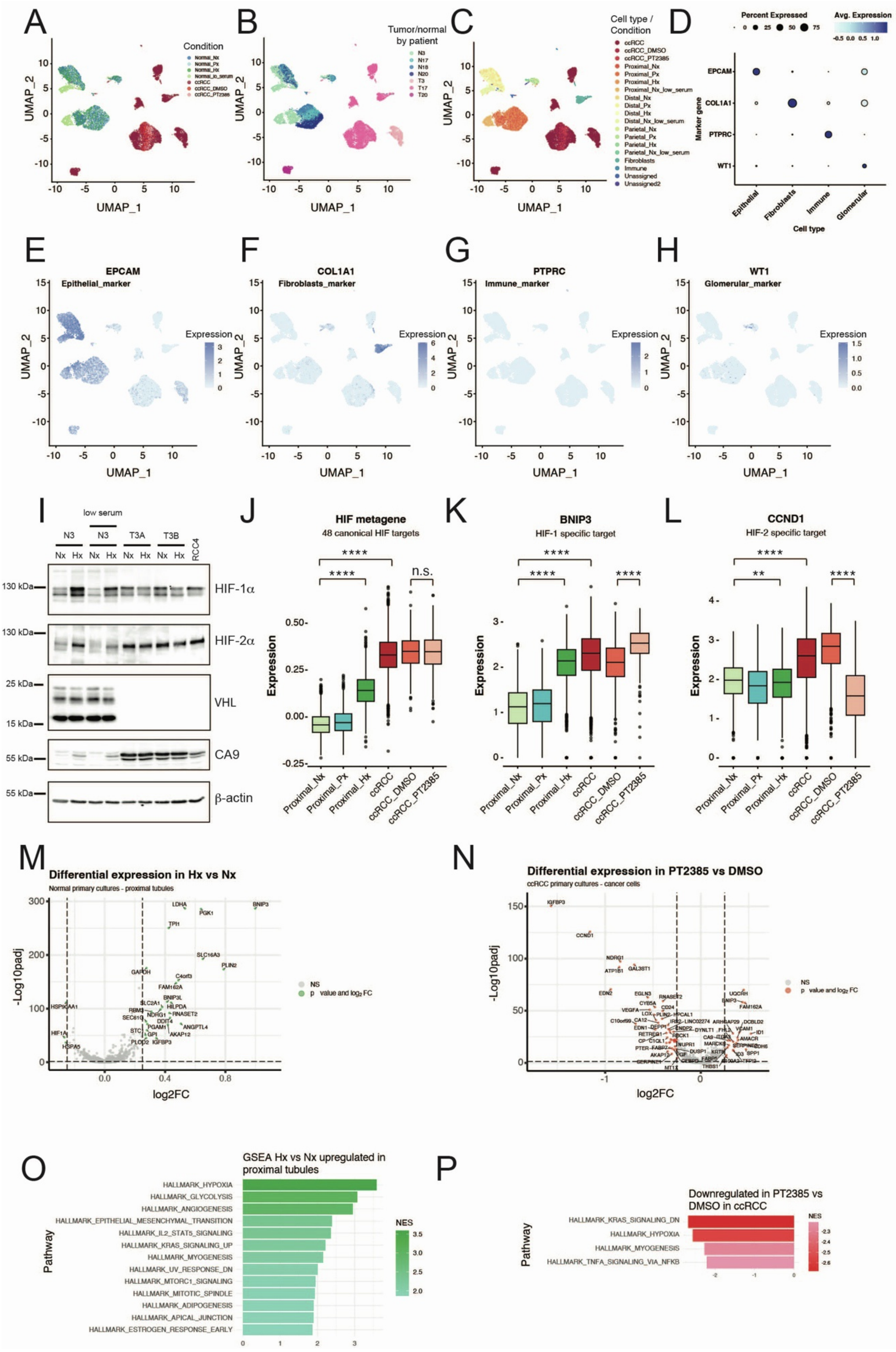
A
B
C
D
E
F
G
H
I
J
K
L
M
N
O
P
Condition
Tumor/normal by patient
Cell type / Condition
Percent Expressed
Avg. Expression
UMAP_1
UMAP_2
EPCAM
Epithelial_marker
COL1A1
Fibroblasts_marker
PTPRC
Immune_marker
WT1
Glomerular_marker
Expression
Marker gene
Cell type
Epithelial
Fibroblasts
Immune
Glomerular
low serum
N3
T3A
T3B
RCC4
Nx
Hx
HIF-1α
HIF-2α
VHL
CA9
β-actin
130 kDa
25 kDa
15 kDa
55 kDa
HIF metagene
48 canonical HIF targets
BNIP3
HIF-1 specific target
CCND1
HIF-2 specific target
n.s.
Proximal_Nx
Proximal_Px
Proximal_Hx
ccRCC
ccRCC_DMSO
ccRCC_PT2385
Differential expression in Hx vs Nx
Normal primary cultures - proximal tubules
Differential expression in PT2385 vs DMSO
ccRCC primary cultures - cancer cells
-Log10padj
log2FC
NS
p value and log2 FC
GSEA Hx vs Nx upregulated in proximal tubules
HALLMARK_HYPOXIA
HALLMARK_GLYCOLYSIS
HALLMARK_ANGIOGENESIS
HALLMARK_EPITHELIAL_MESENCHYMAL_TRANSITION
HALLMARK_IL2_STAT5_SIGNALING
HALLMARK_KRAS_SIGNALING_UP
HALLMARK_MYOGENESIS
HALLMARK_UV_RESPONSE_DN
HALLMARK_MTORC1_SIGNALING
HALLMARK_MITOTIC_SPINDLE
HALLMARK_ADIPOGENESIS
HALLMARK_APICAL_JUNCTION
HALLMARK_ESTROGEN_RESPONSE_EARLY
Pathway
NES
Downregulated in PT2385 vs DMSO in ccRCC
HALLMARK_KRAS_SIGNALING_DN
HALLMARK_HYPOXIA
HALLMARK_MYOGENESIS
HALLMARK_TNFA_SIGNALING_VIA_NFKB

Figure S3

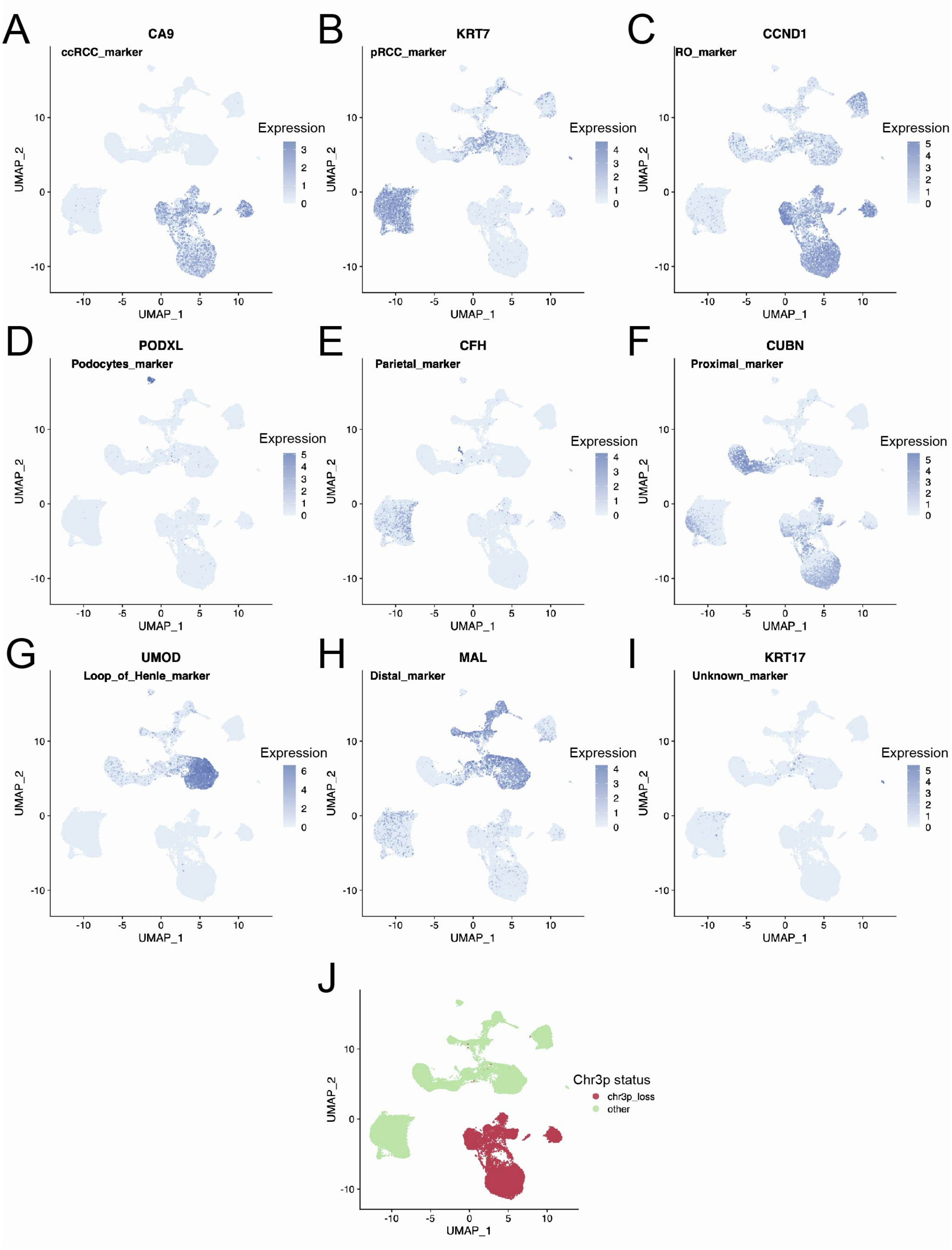
A
CA9
ccRCC_marker
B
KRT7
pRCC_marker
C
CCND1
RO_marker
D
PODXL
Podocytes_marker
E
CFH
Parietal_marker
F
CUBN
Proximal_marker
G
UMOD
Loop_of_Henle_marker
H
MAL
Distal_marker
I
KRT17
Unknown_marker
Expression
UMAP_1
UMAP_2
J
Chr3p status
chr3p_loss
other

Figure S4

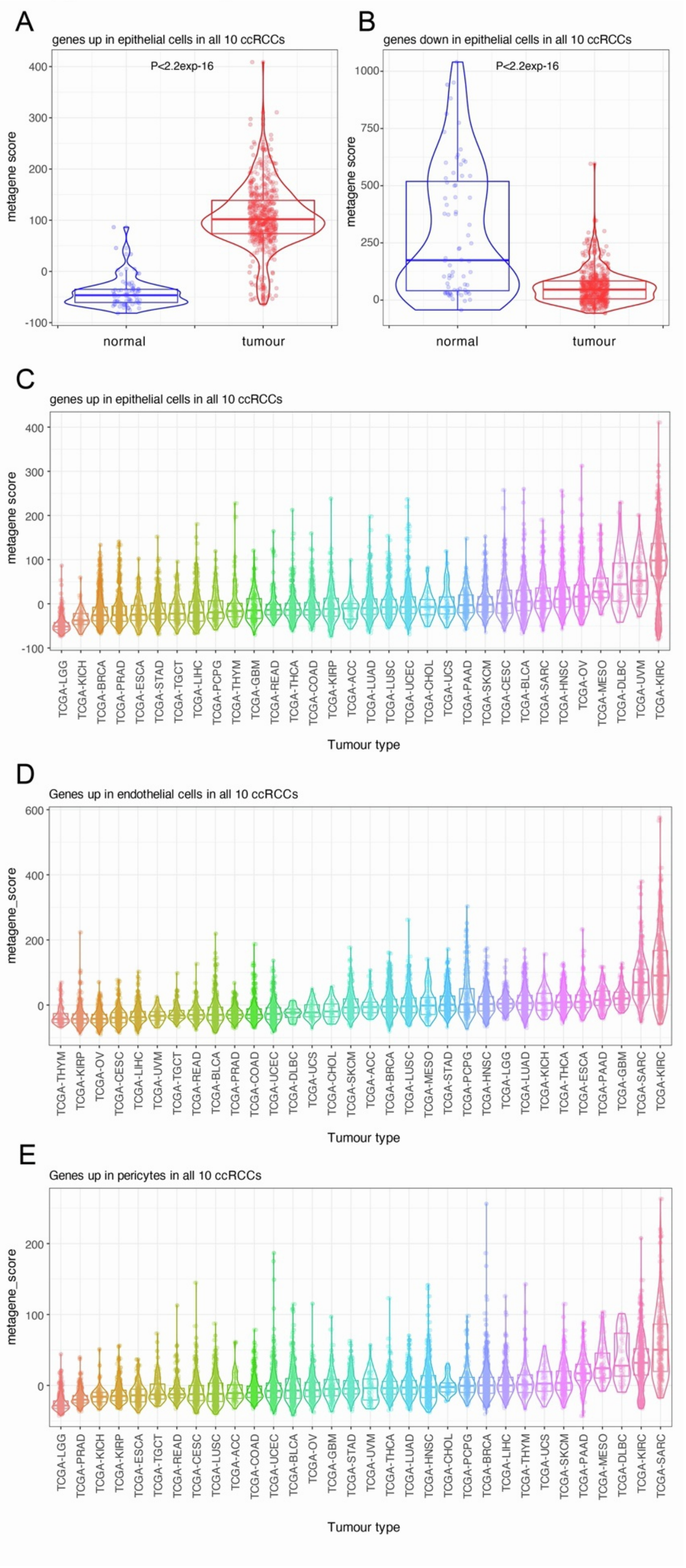
A
genes up in epithelial cells in all 10 ccRCCs
P<2.2exp-16
metagene score
normal
tumour
B
genes down in epithelial cells in all 10 ccRCCs
P<2.2exp-16
metagene score
normal
tumour
C
genes up in epithelial cells in all 10 ccRCCs
metagene score
Tumour type
D
Genes up in endothelial cells in all 10 ccRCCs
metagene_score
Tumour type
E
Genes up in pericytes in all 10 ccRCCs
metagene_score
Tumour type

Figure S5

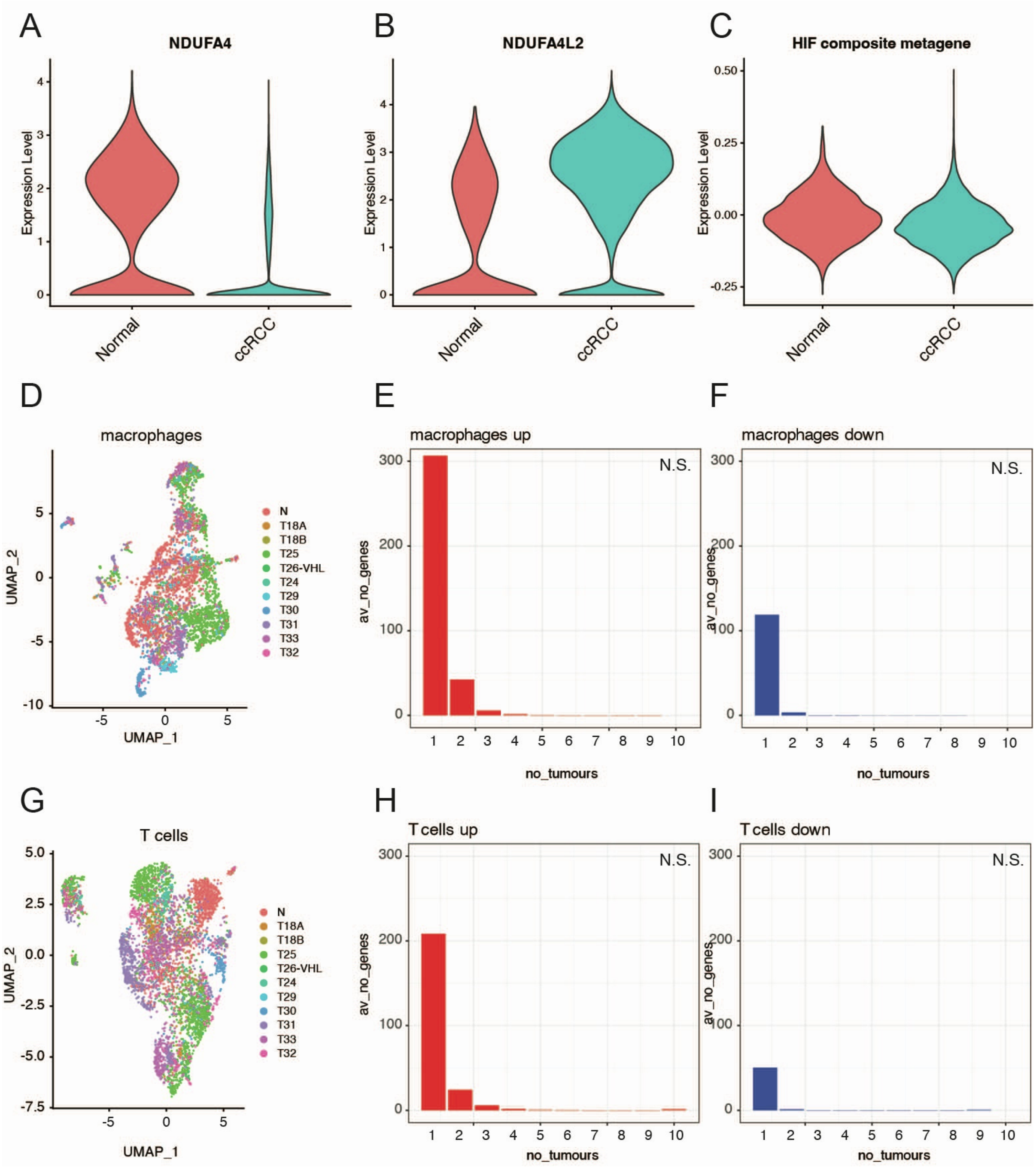

A
NDUFA4
Expression Level
Normal
ccRCC
B
NDUFA4L2
C
HIF composite metagene
D
macrophages
UMAP_2
UMAP_1
N
T18A
T18B
T25
T26-VHL
T24
T29
T30
T31
T33
T32
E
macrophages up
N.S.
av_no_genes
no_tumours
F
macrophages down
G
T cells
H
T cells up
I
T cells down

Figure S6

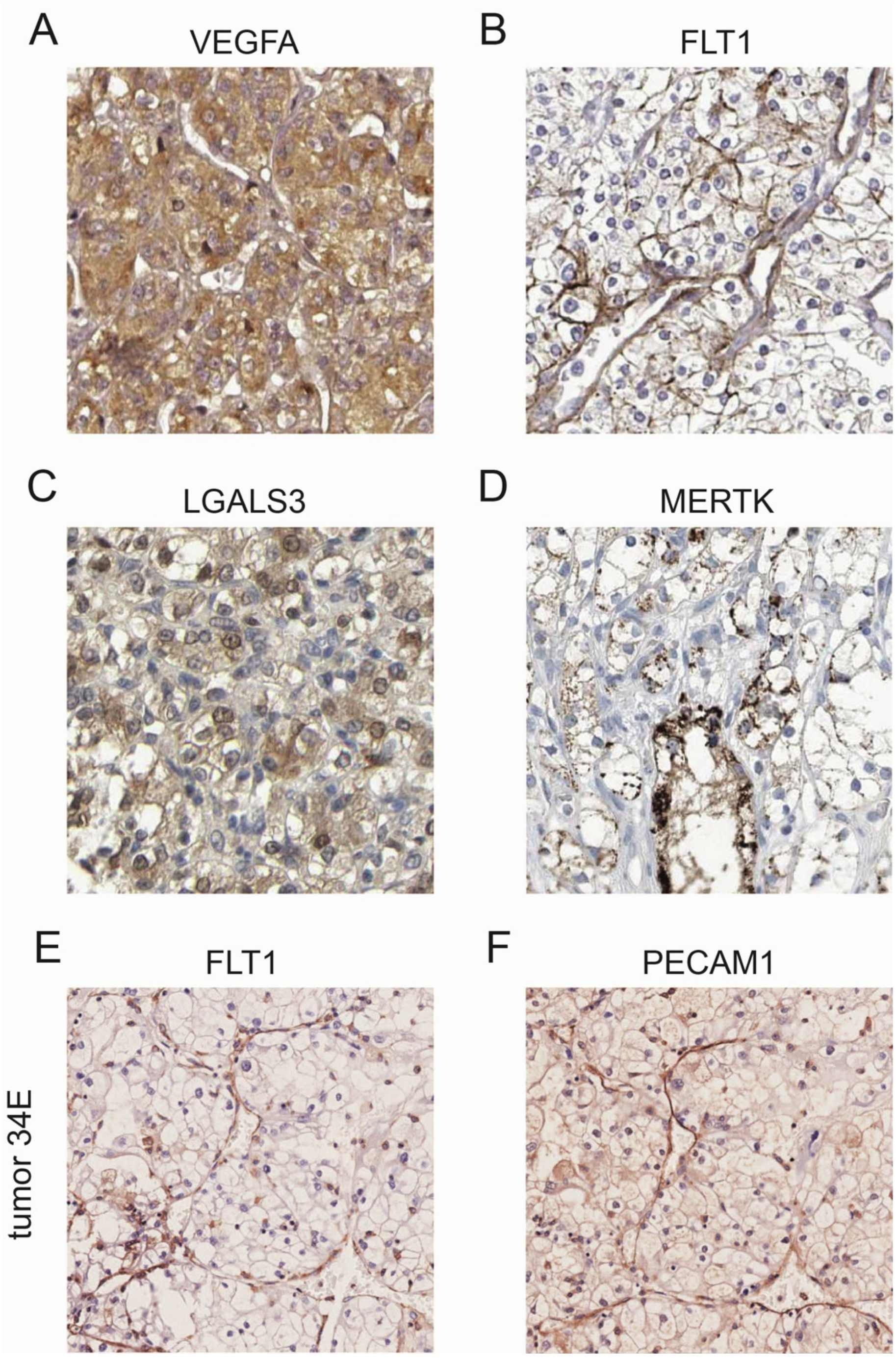

Figure S7

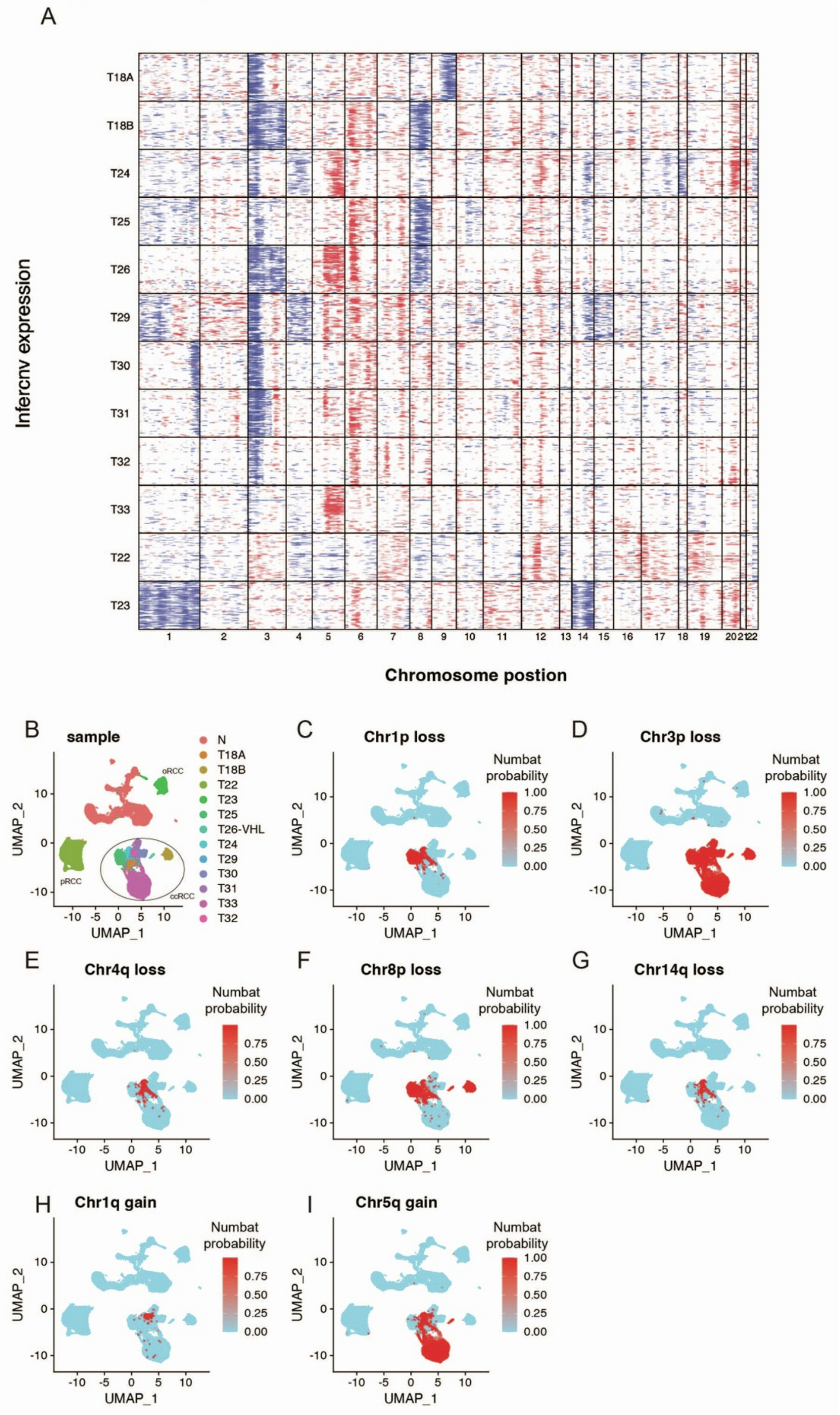
A
Infercnv expression
T18A
T18B
T24
T25
T26
T29
T30
T31
T32
T33
T22
T23
1 2 3 4 5 6 7 8 9 10 11 12 13 14 15 16 17 18 19 20 21 22
Chromosome postion
B sample
N
T18A
T18B
T22
T23
T25
T26-VHL
T24
T29
T30
T31
T33
T32
oRCC
pRCC
ccRCC
C Chr1p loss
D Chr3p loss
E Chr4q loss
F Chr8p loss
G Chr14q loss
H Chr1q gain
I Chr5q gain
Numbat probability
1.00
0.75
0.50
0.25
0.00
UMAP_1
UMAP_2
-10 -5 0 5 10

Figure S8

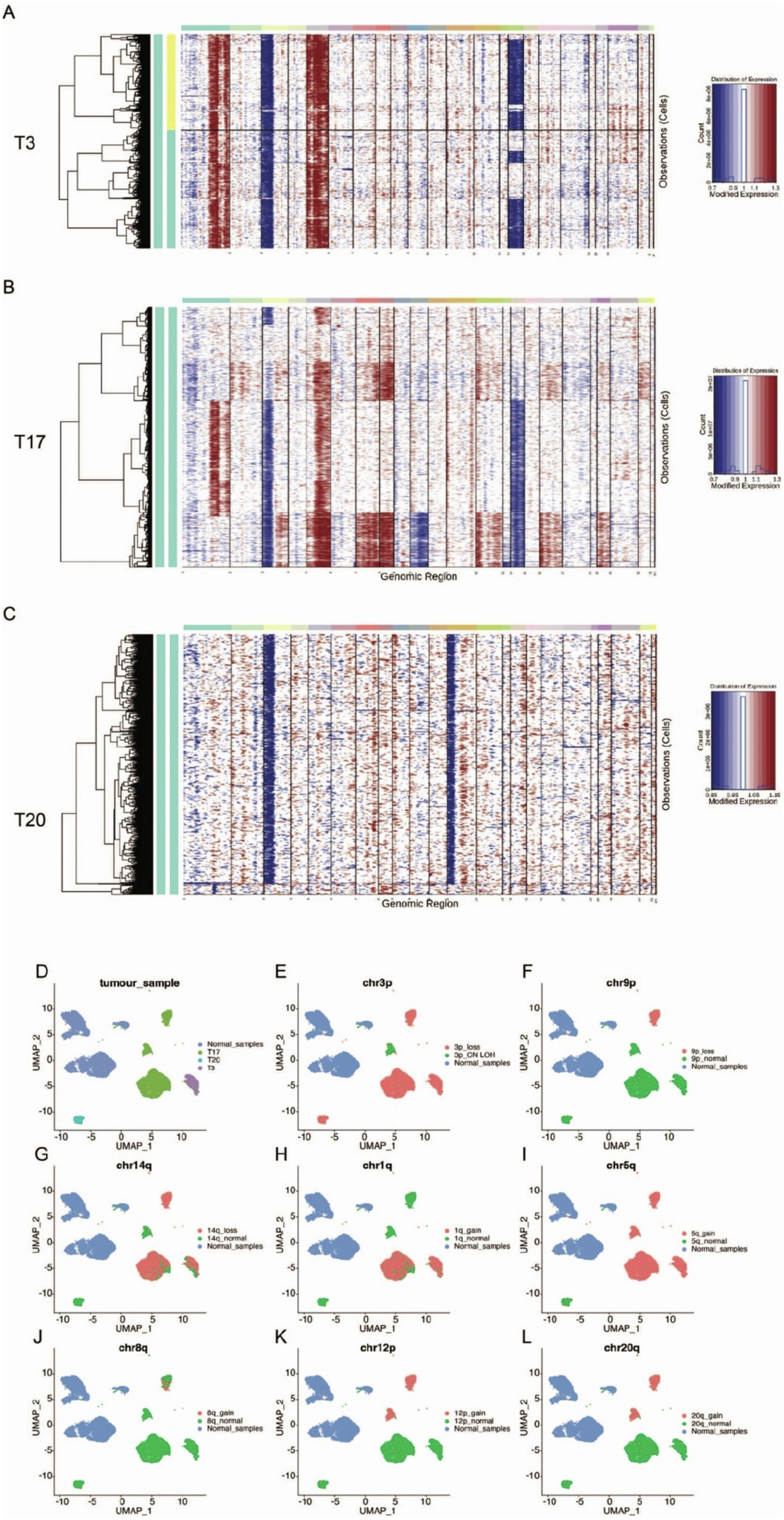
A
T3
Observations (Cells)
Distribution of Expression
Count
Modified Expression
B
T17
Observations (Cells)
Genomic Region
Distribution of Expression
Count
Modified Expression
C
T20
Observations (Cells)
Genomic Region
Distribution of Expression
Count
Modified Expression
D
tumour_sample
Normal_samples
T17
T20
T3
UMAP_1
UMAP_2
E
chr3p
3p_loss
3p_CN LOH
Normal_samples
F
chr9p
9p_loss
9p_normal
Normal_samples
G
chr14q
14q_loss
14q_normal
Normal_samples
H
chr1q
1q_gain
1q_normal
Normal_samples
I
chr5q
5q_gain
5q_normal
Normal_samples
J
chr8q
8q_gain
8q_normal
Normal_samples
K
chr12p
12p_gain
12p_normal
Normal_samples
L
chr20q
20q_gain
20q_normal
Normal_samples

Figure S9

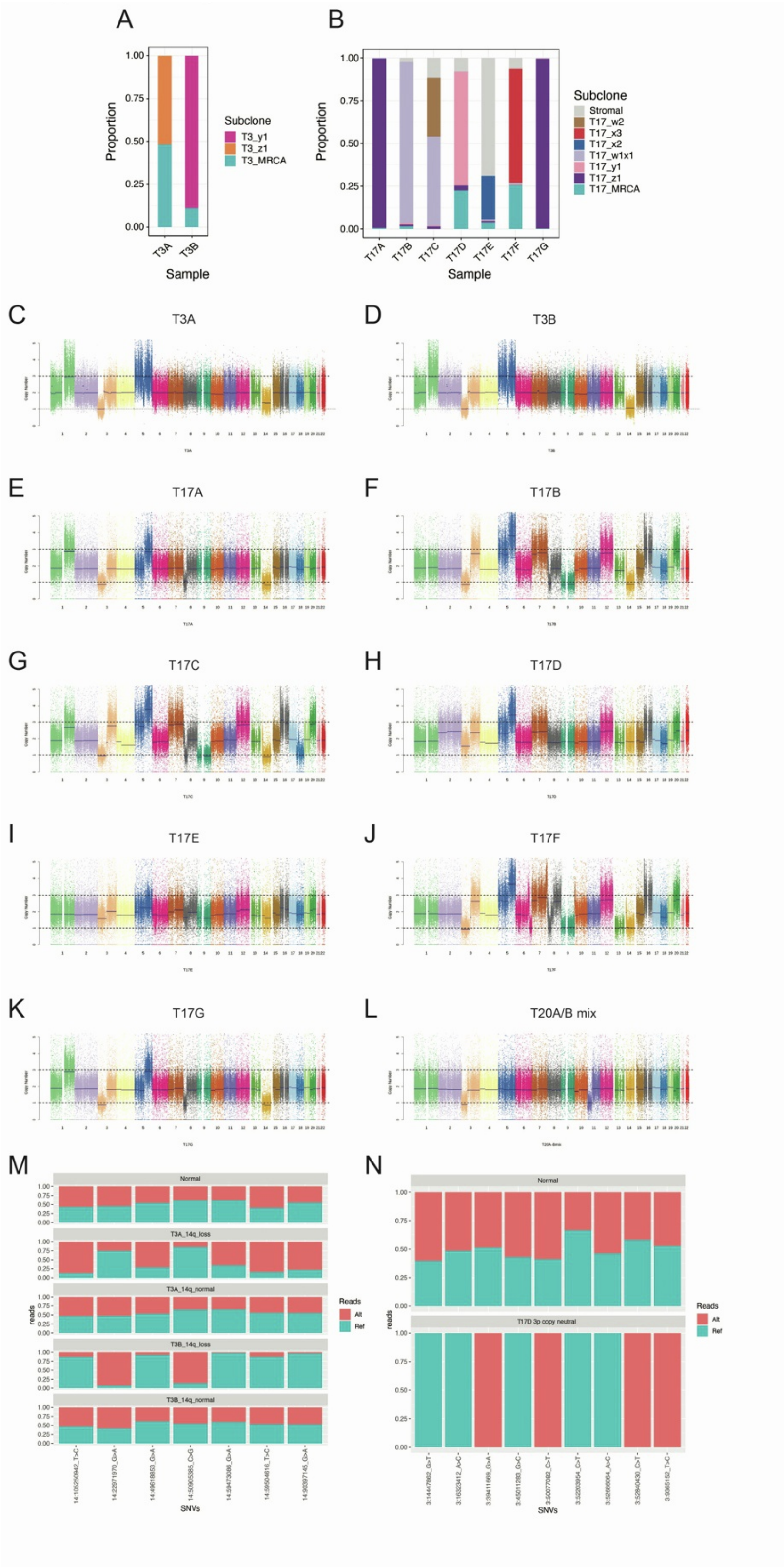
A
B
Proportion
Sample
Subclone
T3_y1
T3_z1
T3_MRCA
T3A
T3B
Stromal
T17_w2
T17_x3
T17_x2
T17_w1x1
T17_y1
T17_z1
T17_MRCA
T17A
T17B
T17C
T17D
T17E
T17F
T17G
C
T3A
D
T3B
E
T17A
F
T17B
G
T17C
H
T17D
I
T17E
J
T17F
K
T17G
L
T20A/B mix
M
N
Normal
T3A_14q_loss
T3A_14q_normal
T3B_14q_loss
T3B_14q_normal
T17D 3p copy neutral
reads
Reads
Alt
Ref
SNVs

Figure S10

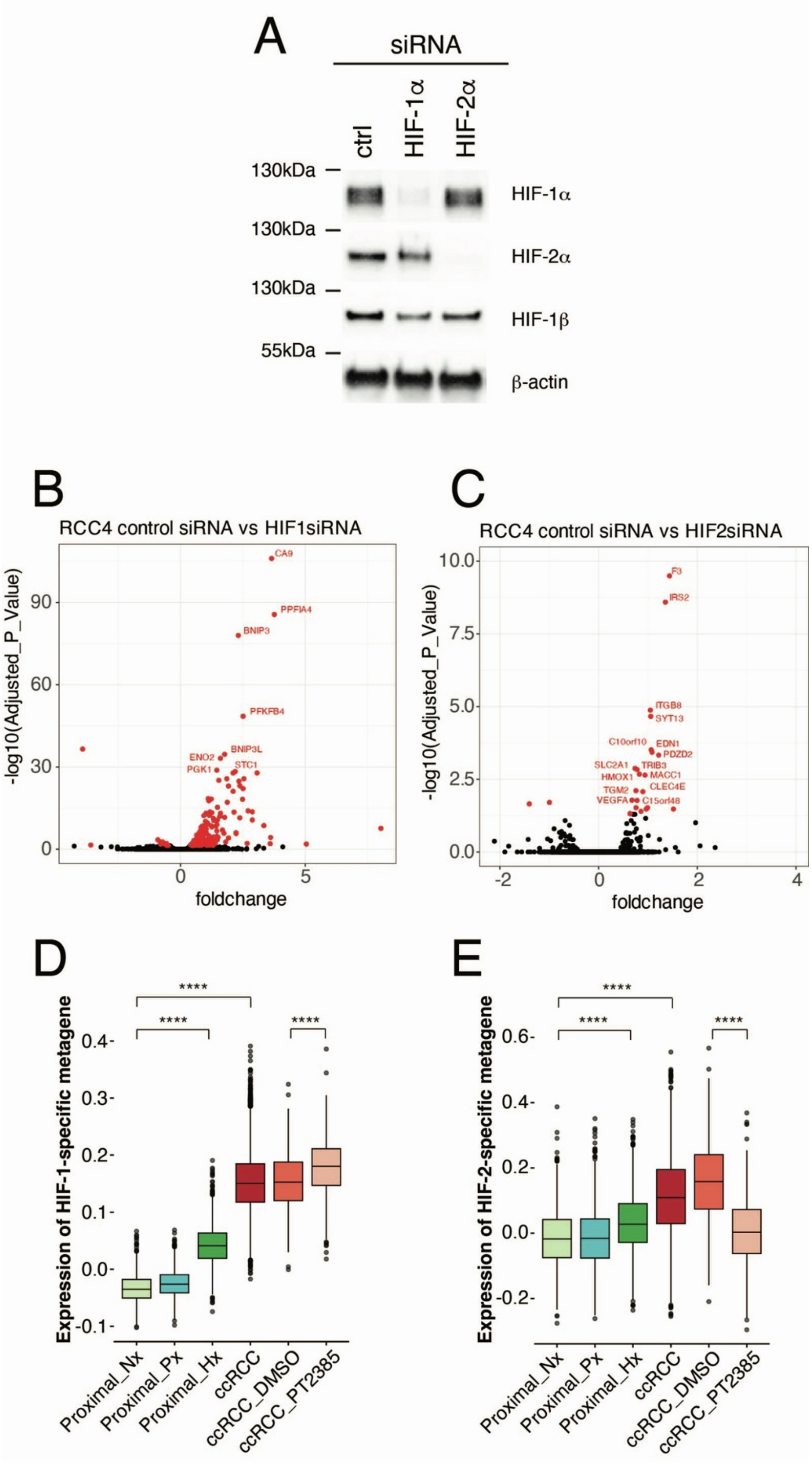

A
siRNA
ctrl
HIF-1α
HIF-2α
130kDa
130kDa
130kDa
55kDa
HIF-1α
HIF-2α
HIF-1β
β-actin
B
RCC4 control siRNA vs HIF1siRNA
-log10(Adjusted_P_Value)
foldchange
CA9
PPFIA4
BNIP3
PFKFB4
BNIP3L
ENO2
PGK1
STC1
C
RCC4 control siRNA vs HIF2siRNA
-log10(Adjusted_P_Value)
foldchange
F3
IRS2
ITGB8
SYT13
C10orf10
EDN1
PDZD2
SLC2A1
TRIB3
HMOX1
MACC1
TGM2
CLEC4E
VEGFA
C15orf48
D
Expression of HIF-1-specific metagene
****
Proximal_Nx
Proximal_Px
Proximal_Hx
ccRCC
ccRCC_DMSO
ccRCC_PT2385
E
Expression of HIF-2-specific metagene
****
Proximal_Nx
Proximal_Px
Proximal_Hx
ccRCC
ccRCC_DMSO
ccRCC_PT2385

Figure S11

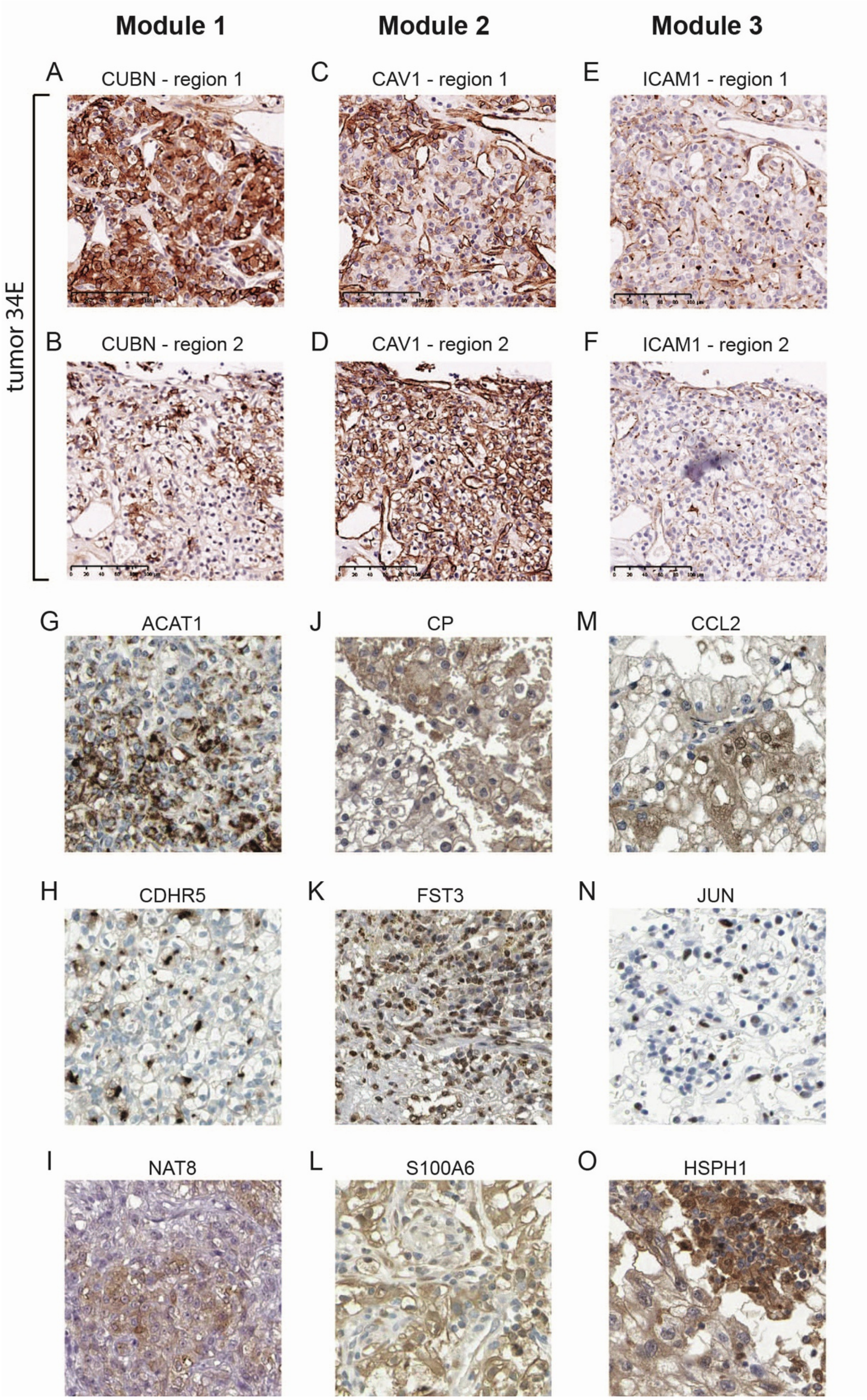

Module 1
Module 2
Module 3
A
CUBN - region 1
C
CAV1 - region 1
E
ICAM1 - region 1
tumor 34E
B
CUBN - region 2
D
CAV1 - region 2
F
ICAM1 - region 2
G
ACAT1
J
CP
M
CCL2
H
CDHR5
K
FST3
N
JUN
I
NAT8
L
S100A6
O
HSPH1

Figure S12

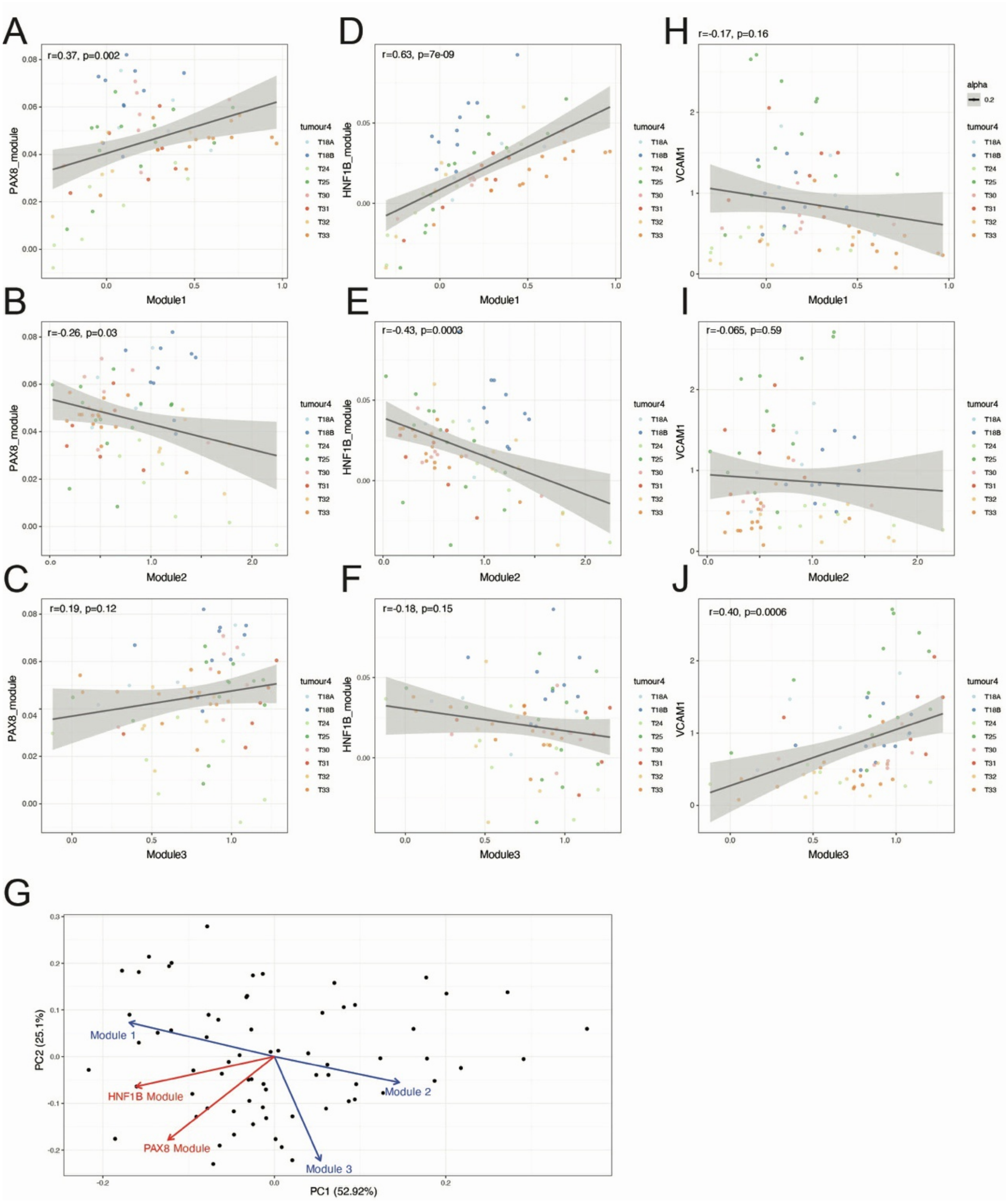
A
r=0.37, p=0.002
PAX8_module
Module1
D
r=0.63, p=7e-09
HNF1B_module
Module1
H
r=-0.17, p=0.16
VCAM1
Module1
alpha
0.2
tumour4
T18A
T18B
T24
T25
T30
T31
T32
T33
B
r=-0.26, p=0.03
PAX8_module
Module2
E
r=-0.43, p=0.0003
HNF1B_module
Module2
I
r=-0.065, p=0.59
VCAM1
Module2
C
r=0.19, p=0.12
PAX8_module
Module3
F
r=-0.18, p=0.15
HNF1B_module
Module3
J
r=0.40, p=0.0006
VCAM1
Module3
G
Module 1
Module 2
Module 3
HNF1B Module
PAX8 Module
PC2 (25.1%)
PC1 (52.92%)

Figure S13

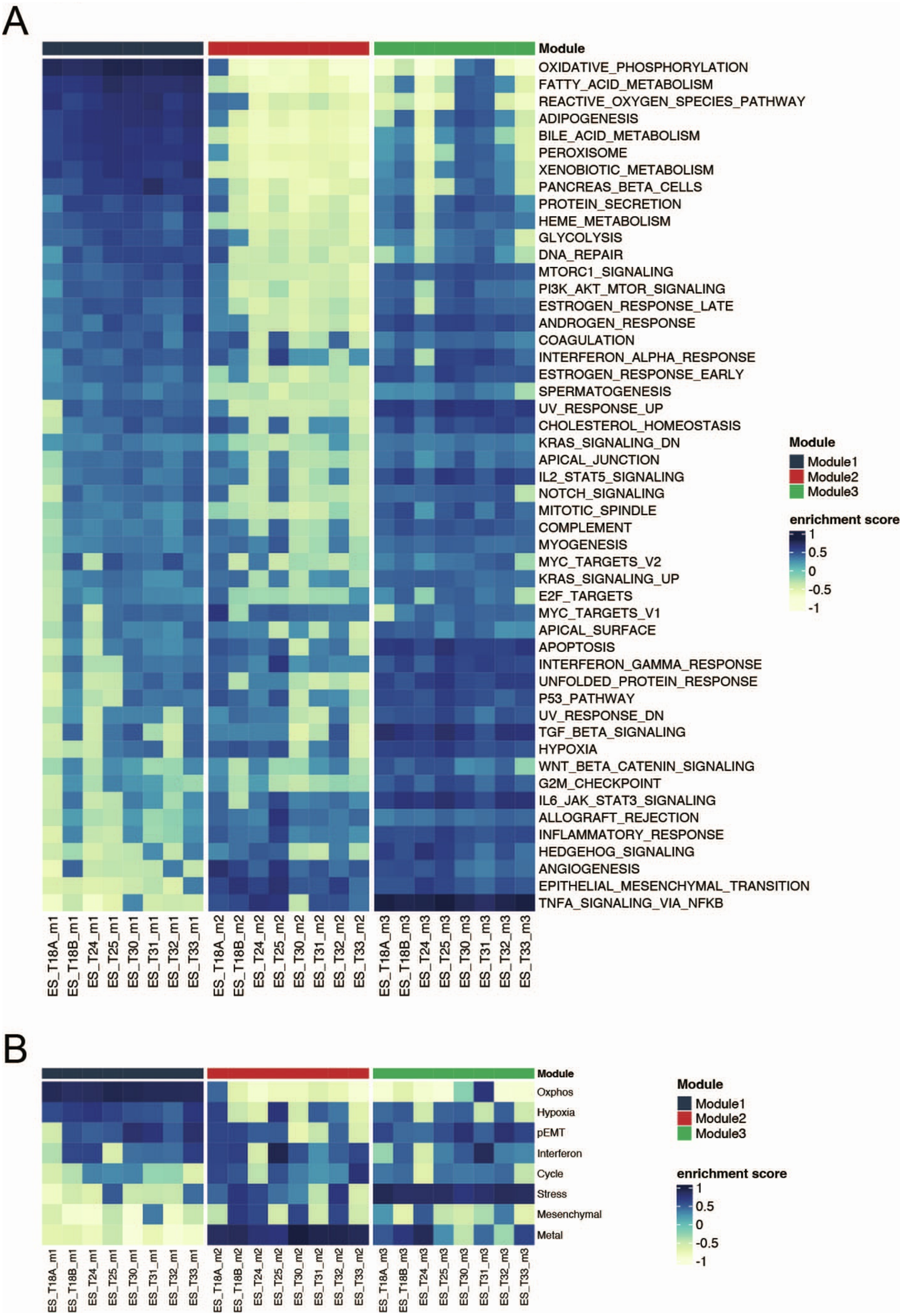

A
Module
OXIDATIVE_PHOSPHORYLATION
FATTY_ACID_METABOLISM
REACTIVE_OXYGEN_SPECIES_PATHWAY
ADIPOGENESIS
BILE_ACID_METABOLISM
PEROXISOME
XENOBIOTIC_METABOLISM
PANCREAS_BETA_CELLS
PROTEIN_SECRETION
HEME_METABOLISM
GLYCOLYSIS
DNA_REPAIR
MTORC1_SIGNALING
PI3K_AKT_MTOR_SIGNALING
ESTROGEN_RESPONSE_LATE
ANDROGEN_RESPONSE
COAGULATION
INTERFERON_ALPHA_RESPONSE
ESTROGEN_RESPONSE_EARLY
SPERMATOGENESIS
UV_RESPONSE_UP
CHOLESTEROL_HOMEOSTASIS
KRAS_SIGNALING_DN
APICAL_JUNCTION
IL2_STAT5_SIGNALING
NOTCH_SIGNALING
MITOTIC_SPINDLE
COMPLEMENT
MYOGENESIS
MYC_TARGETS_V2
KRAS_SIGNALING_UP
E2F_TARGETS
MYC_TARGETS_V1
APICAL_SURFACE
APOPTOSIS
INTERFERON_GAMMA_RESPONSE
UNFOLDED_PROTEIN_RESPONSE
P53_PATHWAY
UV_RESPONSE_DN
TGF_BETA_SIGNALING
HYPOXIA
WNT_BETA_CATENIN_SIGNALING
G2M_CHECKPOINT
IL6_JAK_STAT3_SIGNALING
ALLOGRAFT_REJECTION
INFLAMMATORY_RESPONSE
HEDGEHOG_SIGNALING
ANGIOGENESIS
EPITHELIAL_MESENCHYMAL_TRANSITION
TNFA_SIGNALING_VIA_NFKB
ES_T18A_m1
ES_T18B_m1
ES_T24_m1
ES_T25_m1
ES_T30_m1
ES_T31_m1
ES_T32_m1
ES_T33_m1
ES_T18A_m2
ES_T18B_m2
ES_T24_m2
ES_T25_m2
ES_T30_m2
ES_T31_m2
ES_T32_m2
ES_T33_m2
ES_T18A_m3
ES_T18B_m3
ES_T24_m3
ES_T25_m3
ES_T30_m3
ES_T31_m3
ES_T32_m3
ES_T33_m3
Module
Module1
Module2
Module3
enrichment score
1
0.5
0
-0.5
-1
B
Module
Oxphos
Hypoxia
pEMT
Interferon
Cycle
Stress
Mesenchymal
Metal
ES_T18A_m1
ES_T18B_m1
ES_T24_m1
ES_T25_m1
ES_T30_m1
ES_T31_m1
ES_T32_m1
ES_T33_m1
ES_T18A_m2
ES_T18B_m2
ES_T24_m2
ES_T25_m2
ES_T30_m2
ES_T31_m2
ES_T32_m2
ES_T33_m2
ES_T18A_m3
ES_T18B_m3
ES_T24_m3
ES_T25_m3
ES_T30_m3
ES_T31_m3
ES_T32_m3
ES_T33_m3
Module
Module1
Module2
Module3
enrichment score
1
0.5
0
-0.5
-1

Figure S14

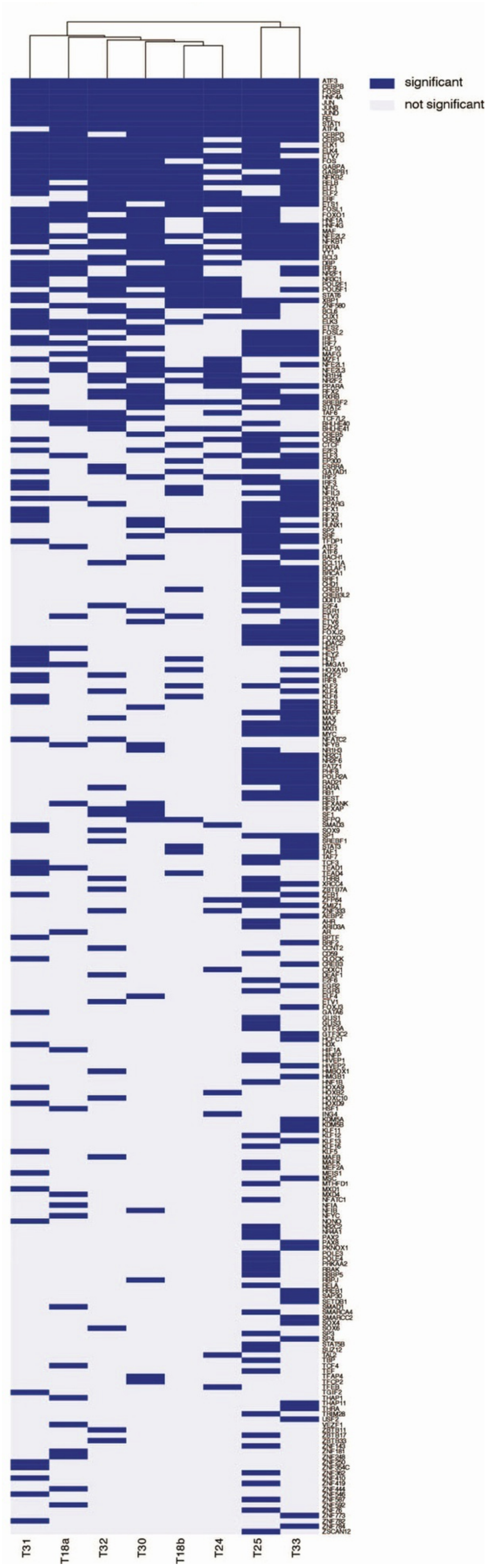
significant
not significant
T31
T18a
T32
T30
T18b
T24
T25
T33

Figure S15

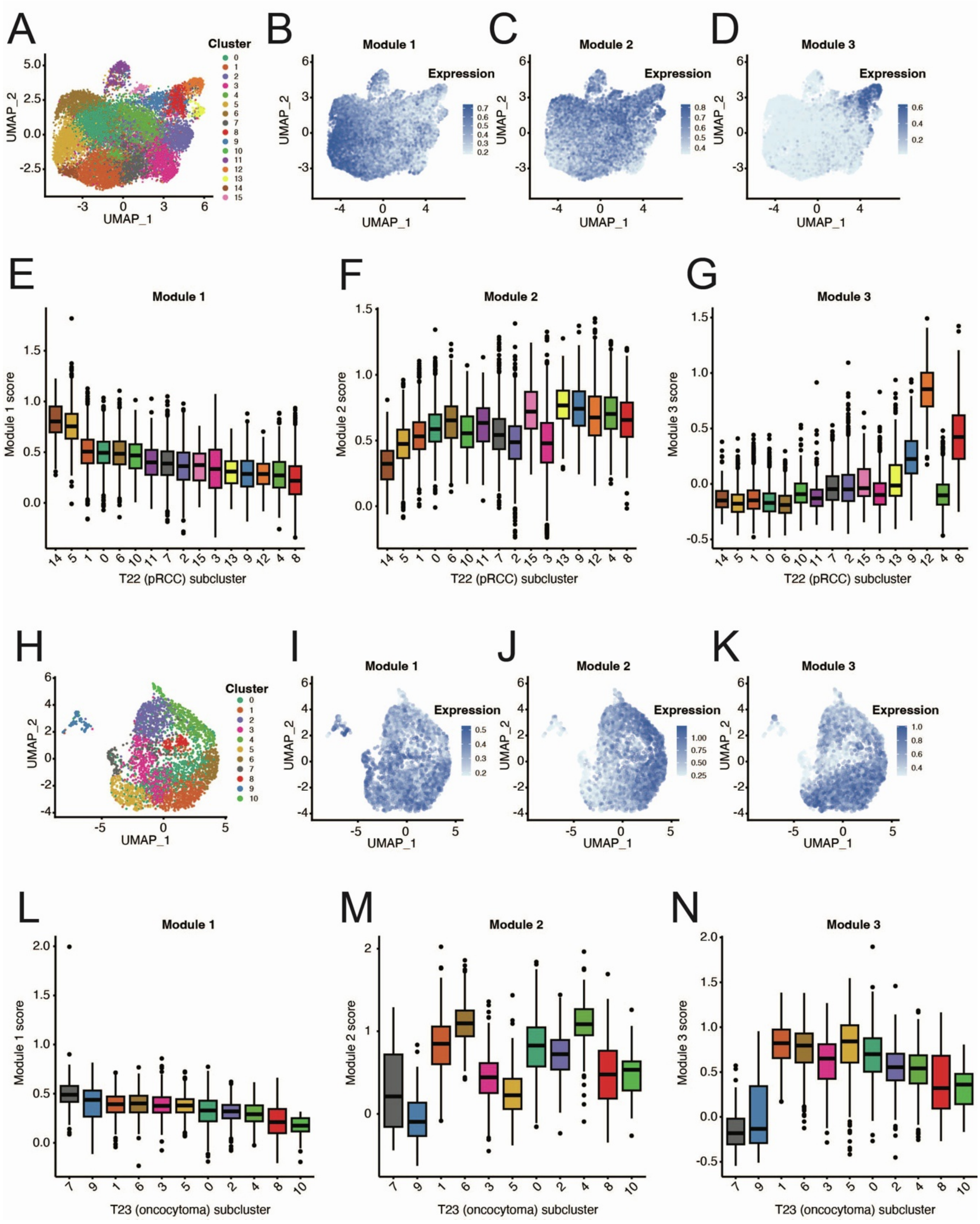
A
Cluster
UMAP_1
UMAP_2
B
Module 1
Expression
C
Module 2
Expression
D
Module 3
Expression
E
Module 1
Module 1 score
T22 (pRCC) subcluster
F
Module 2
Module 2 score
T22 (pRCC) subcluster
G
Module 3
Module 3 score
T22 (pRCC) subcluster
H
Cluster
I
Module 1
Expression
J
Module 2
Expression
K
Module 3
Expression
L
Module 1
Module 1 score
T23 (oncocytoma) subcluster
M
Module 2
Module 2 score
T23 (oncocytoma) subcluster
N
Module 3
Module 3 score
T23 (oncocytoma) subcluster

Supplemental Figure Legends

Figure S1. scRNA-seq analysis of freshly-dissociated biopsy samples, Related to Figure 1. UMAP plots of cells from freshly-biopsied tumor and background kidney biopsy samples showing (A) samples from different tumor types, normal background kidney and cystic background kidney; (B) sample type and patient; and expression of (C) EPCAM, (D) PECAM1, (E) ACTA2, (F) PTPRC, and (G) WT1. (H) Dotplots for cells from freshly-biopsied samples showing marker gene expression according to major cell type. Plots showing cellular frequency distribution against between-patient cell mixing score, stratified by cell type, for (I) normal and (J) tumor samples. (K) Box-and-whisker plots showing proportion of each cell type in normal and tumor samples. Box-and-whisker plots show median, inter-quartile range and range. See also Figure 1.

Figure S2. scRNA-seq analysis of cultured cells, Related to Figure 1. UMAP plots of cells from cultured tumor and normal kidney samples showing (A) sample type and intervention; (B) sample type and patient; and (C) cell type and intervention. (D) Dotplots for cells from primary cultures showing marker gene expression according to major cell type. UMAP plots showing expression of (E) EPCAM, (F) COL1A1, (G) PTPRC, and (H) WT1. (I) Immunoblots showing levels of HIF-1α, HIF-2α, VHL, CA9 and β-actin (loading control) protein in patient 3 cultured cells incubated in normoxia (Nx), hypoxia (Hx, 0.5% O_2), or low serum (0.5% FBS) conditions for 16 hours. Depicts hypoxic-induction of HIF-1α, HIF-2α and CA9 in primary normal kidney cultures, yet constitutive expression in primary VHL-deficient ccRCC cultures. RCC4 (established ccRCC cell line) were used as a reference. Violin plots showing RNA expression of (J) composite HIF metagene[75], (K) BNIP3 and (L) CCND1 in single cells cultured in normoxia or 0.5% hypoxia or with DMSO or 1μM PT2385 (Belzutifan, HIF-2 inhibitor) for 16 hours. n.s. not significant, **p<0.01, ****p<0.0001. (M) Volcano plot showing genes differentially expressed in normal proximal tubular cells cultured in 0.5% hypoxia vs normoxia for 16 hours. Green denotes genes with log2(fold-change) >0.25 or <-0.25 and Bonferroni-adjusted-p-value <0.05; remaining genes colored grey. (N) Volcano plot showing genes differentially expressed in ccRCC epithelial cells cultured with 1μM PT2385 for 16 hours. Red denotes genes with log2(fold-change)>0.25 or <-0.25 and Bonferroni-adjusted-p-value<0.05; remaining genes colored grey. (O) GSEA showing Hallmark pathways enriched amongst genes induced by hypoxia in normal proximal tubular cells. (P) GSEA showing Hallmark pathways enriched amongst PT2385-downregulated genes in ccRCC epithelial cells. Box-and-whisker plots show median, inter-quartile range and range. See also Figure 1.

Figure S3. Marker gene and genetic marker validation of epithelial cell types, Related to Figure 2. UMAP plots of epithelial cells from freshly-biopsied samples showing expression of (A) CA9, (B) KRT7, (C) CCND1, (D) PODXL, (E) CFH, (F) CUBN, (G) UMOD, (H) MAL, (I) KRT17, (J) chromosome 3p copy number loss. See also Figure 2.

Figure S4. Analysis of composite metagenes, based on scRNA-seq analysis, in bulk RNA-seq analysis from the TCGA cohort, Related to Figure 2. (A) Violin and box-and-whisker plots showing collective expression of genes upregulated in all 10 ccRCCs vs proximal tubule cells (as a composite metagene) shown in bulk RNA data from normal and tumor samples from the TCGA-KIRC cohort. (B) Same analysis for genes downregulated in all 10 ccRCCs vs proximal tubule cells. Violin and box-and-whisker plots showing expression of composite

metagenes based on genes up regulated in (C) epithelial cells (D) endothelial cells and (E) pericytes from all 10 ccRCCs in bulk RNA-seq analysis of tumor samples from 32 tumor types in the TCGA cohort. Tumor types are ranked according to their median expression of each metagene. Box-and-whisker plots show median, inter-quartile range and range. See also Figure 2.

Figure S5. Analysis of mitochondrial subunit switching in pericytes and conserved patterns of gene dysregulation in macrophages and T cell populations, Related to Figure 3. Violin plots showing expression of (A) NDUFA4, (B) NDUFA4L2, and (C) composite HIF-target metagene[75] in pericytes derived from freshly-biopsied normal kidney samples (red) and ccRCC samples (blue). (D) UMAP plot of macrophages from freshly-biopsied samples showing sample type and patient. (E) Intersection analysis showing the overlap between genes upregulated in macrophages from each of the 10 ccRCC tumors compared to macrophages from the normal kidney samples. (F) The same analysis for downregulated genes. (G) UMAP plot of T cells from freshly-biopsied samples showing sample type and patient. (H) Intersection analysis showing the overlap between genes upregulated in T cells from each of the 10 ccRCC tumors compared to T cells from the normal kidney samples. (I) The same analysis for downregulated genes. See also Figure 3.

Figure S6. Immunostaining of representative ligand-receptor pairings, Related to Figure 3. (A) VEGFA, (B) FLT1, (C) LGALS3 and (D) MERTK protein expression in ccRCC tumors from the Human Protein Atlas. Serial sections from tumor 34E showing co-localization of (E) FLT1 and (F) the endothelial marker PECAM1. See also Figure 3.

Figure S7. Analysis of chromosomal copy number variation (CNV) in freshly-biopsied samples, Related to Figure 4. (A) Heatmap showing InferCNV-normalized gene expression in freshly-biopsied tumor epithelial cells compared to normal epithelial cells across chromosomal regions (red = increased expression, blue = reduced expression). Cells are randomly downsampled to 100 cells per tumor. UMAP plots of epithelial cells from freshly-biopsied samples showing (B) sample type, and NUMBAT calculated probability of (C) chromosome 1p loss, (D) chromosome 3p loss, (E) chromosome 4q loss, (F) chromosome 8p loss, (G) chromosome 14q loss, (H) chromosome 1q gain and (I) chromosome 5q gain. See also Figure 4.

Figure S8. Analysis of chromosomal copy number variation (CNV) in cultured samples, Related to Figure 4. Heatmaps showing gene expression in cultured tumor sample epithelial cells compared to normal epithelial cells across chromosomal regions (red = increased expression - predicted copy number gain, blue = reduced expression – predicted copy number loss) for tumors (A) T3, (B) T17 and (C) T20. Each column represents a different chromosomal position and each row represents an individual cell. UMAP plots of cells from cultured samples showing (D) sample type, and InferCNV identification of (E) chromosome 3p loss, (F) chromosome 9p loss, (G) chromosome 14q loss, (H) chromosome 1q gain, (I) chromosome 5q gain, (J) chromosome 8q gain, (K) chromosome 12p gain and (L) chromosome 20q gain. One subclone of T17 exhibited copy neutral loss of heterozygosity of chromosome 3p (E). See also Figure 4.

Figure S9. Analysis of chromosomal copy number and loss of heterozygosity, Related to Figure 4. Stacked bar charts showing clonal and stromal composition of samples from (A) T3 and (B) T17 as determined by scRNA-seq, demonstrating how bulk exome-seq on the same samples could be used to map additional genetic information onto subclones. CNVkit analysis

of chromosomal copy number in bulk exome-seq data from (C) T3A, (D) T3B, (E) T17A, (F) T17B, (G) T17C, (H) T17D, (I) T17E, (J) T17F, (K) T17G, (L) T20 mix of samples A and B. (M) scRNA-seq allele frequency for germline heterozygous SNPs on chromosome 14q in cancer cells from T3A and T3B samples stratified by 14q (loss) status, showing independent loss of opposing alleles. (N) scRNA-seq allele frequency for germline heterozygous SNPs on chromosome 3p in a subcluster of cancer cells from sample T17D that was 3p diploid, demonstrating expression of single alleles and therefore copy neutral loss of heterozygosity. See also Figure 4.

Figure S10. Bulk RNA-seq analysis of HIF-1 and HIF-2 dependent genes in RCC4 cells, Related to Figure 4. (A) Western blots showing HIF-1α, HIF-2α, HIF-1β and β-actin (loading control) protein levels in RCC4 cells treated with control, HIF-1α or HIF-2α siRNAs. Volcano plots showing log2 fold-change and -log10 adjusted p-values for (B) HIF-1a siRNA versus control siRNA and (C) HIF-2a siRNA versus control siRNA in bulk RNA-seq analysis of RCC4 cells. Red denotes genes with an adjusted p-value of <0.05; all other genes colored in black. Box-and-whisker plots showing expression of (D) HIF-1-specific metagene and (E) HIF-2-specific metagene in scRNA-seq analysis of cultured samples, demonstrating induction of both metagenes by hypoxia in proximal tubules, overexpression of both metagenes in ccRCC epithelial cells vs proximal tubules, and specific downregulation of the HIF-2-specific metagene by the HIF-2 inhibitor. **** $p<0.0001$. Box-and-whisker plots show median, inter-quartile range and range. See also Figure 4.

Figure S11. Immunostaining of representative module 1, 2 and 3 genes in ccRCC tumor, Related to Figure 5. Immunostaining of (A, B) CUBN, (C, D) CAV1 and (E, F) ICAM1 in two regions of tumor sample 34E. Expression of (G) ACAT1, (H) CDHR5, (I) NAT8, (J) CP, (K) FST3, (L) S100A6, (M) CCL2, (N) JUN, and (O) HSPH1 in ccRCC samples from the Human Protein Atlas. See also Figure 5.

Figure S12. Correlation of gene modules with PAX8 and HNF1b gene programs. (A-E), Related to Figure 5. Scatter plots showing mean expression of composite metagenes based on Module 1,2 and 3 genes and the 100 most PAX8- and HNF1b-dependent genes from Patel et al in tumor cell clusters from fresh biopsy samples. (G) Biplot showing PCA analysis of expression of these 5 gene modules across the individual tumor clusters. The arrows represent the original axes for the 5 modules in the first two principal components. See also Figure 5.

Figure S13. Gene set enrichment analysis of genes correlating with modules 1, 2 and 3, Related to Figure 5. (A) Heatmap showing enrichment scores for Hallmark pathway genes amongst genes correlating with Module 1, 2 and 3 genes in each of the 8 ccRCC tumors studied. Pathways are ordered by the average Module-1-correlating enrichment score across tumors. (B) Heatmap showing enrichment scores for Barkley genesets amongst genes correlating with Module 1, 2 and 3 genes. Genesets are ordered by the average Module-1-correlating enrichment scores across tumors. See also Figure 5.

Figure S14. Heatmap showing heterogenous transcriptional programs in individual tumors, Related to Figure 5. SCENIC analysis was used to identify transcription factors whose activity exhibited significant intratumor heterogeneity (dark blue) in each of the 8 tumors. See also Figure 5.

Figure S15. Expression of Module 1, 2 and 3 genes in pRCC and oncocytoma tumors, Related to Figure 7. UMAP plots showing (A) cell clusters, and (B) Module 1, (C) Module 2

and (D) Module 3 metagene expression in pRCC (T22) epithelial cells. (E-G) Box-and-whisker plots showing T22 cluster-level composite module scores for modules 1, 2 and 3 in clusters arranged in decreasing order of median Module 1 score. UMAP plots showing (H) cell clusters, and (I) Module 1, (J) Module 2 and (K) Module 3 metagene expression in oncocytoma (T23) epithelial cells. (L-M) Box-and-whisker plots showing T23 cluster-level composite module scores for modules 1, 2 and 3 in clusters arranged in decreasing order of median Module 1 score. Box-and-whisker plots show median, inter-quartile range and range. See also Figure 7.
